# Hepatectomy-Induced Alterations in Hepatic Perfusion and Function - Toward Multi-Scale Computational Modeling for a Better Prediction of Post-hepatectomy Liver Function

**DOI:** 10.3389/fphys.2021.733868

**Published:** 2021-11-18

**Authors:** Bruno Christ, Maximilian Collatz, Uta Dahmen, Karl-Heinz Herrmann, Sebastian Höpfl, Matthias König, Lena Lambers, Manja Marz, Daria Meyer, Nicole Radde, Jürgen R. Reichenbach, Tim Ricken, Hans-Michael Tautenhahn

**Affiliations:** ^1^Cell Transplantation/Molecular Hepatology Lab, Department of Visceral, Transplant, Thoracic and Vascular Surgery, University of Leipzig Medical Center, Leipzig, Germany; ^2^RNA Bioinformatics and High-Throughput Analysis, Faculty of Mathematics and Computer Science, Friedrich Schiller University Jena, Jena, Germany; ^3^Optisch-Molekulare Diagnostik und Systemtechnologié, Leibniz Institute of Photonic Technology (IPHT), Jena, Germany; ^4^InfectoGnostics Research Campus Jena, Jena, Germany; ^5^Experimental Transplantation Surgery, Department of General, Visceral and Vascular Surgery, Jena University Hospital, Jena, Germany; ^6^Medical Physics Group, Institute of Diagnostic and Interventional Radiology, Jena University Hospital, Jena, Germany; ^7^Faculty of Engineering Design, Production Engineering and Automotive Engineering, Institute for Systems Theory and Automatic Control, University of Stuttgart, Stuttgart, Germany; ^8^Systems Medicine of the Liver Lab, Institute for Theoretical Biology, Humboldt-University Berlin, Berlin, Germany; ^9^Faculty of Aerospace Engineering and Geodesy, Institute of Mechanics, Structural Analysis and Dynamics, University of Stuttgart, Stuttgart, Germany; ^10^Department of General, Visceral and Vascular Surgery, Jena University Hospital, Jena, Germany

**Keywords:** multi-scale modeling, liver regeneration, liver surgery, liver perfusion, perfusion/function relationship, mechanoperception, rodent models of liver surgery

## Abstract

Liver resection causes marked perfusion alterations in the liver remnant both on the organ scale (vascular anatomy) and on the microscale (sinusoidal blood flow on tissue level). These changes in perfusion affect hepatic functions via direct alterations in blood supply and drainage, followed by indirect changes of biomechanical tissue properties and cellular function. Changes in blood flow impose compression, tension and shear forces on the liver tissue. These forces are perceived by mechanosensors on parenchymal and non-parenchymal cells of the liver and regulate cell-cell and cell-matrix interactions as well as cellular signaling and metabolism. These interactions are key players in tissue growth and remodeling, a prerequisite to restore tissue function after PHx. Their dysregulation is associated with metabolic impairment of the liver eventually leading to liver failure, a serious post-hepatectomy complication with high morbidity and mortality. Though certain links are known, the overall functional change after liver surgery is not understood due to complex feedback loops, non-linearities, spatial heterogeneities and different time-scales of events. Computational modeling is a unique approach to gain a better understanding of complex biomedical systems. This approach allows (i) integration of heterogeneous data and knowledge on multiple scales into a consistent view of how perfusion is related to hepatic function; (ii) testing and generating hypotheses based on predictive models, which must be validated experimentally and clinically. In the long term, computational modeling will (iii) support surgical planning by predicting surgery-induced perfusion perturbations and their functional (metabolic) consequences; and thereby (iv) allow minimizing surgical risks for the individual patient. Here, we review the alterations of hepatic perfusion, biomechanical properties and function associated with hepatectomy. Specifically, we provide an overview over the clinical problem, preoperative diagnostics, functional imaging approaches, experimental approaches in animal models, mechanoperception in the liver and impact on cellular metabolism, omics approaches with a focus on transcriptomics, data integration and uncertainty analysis, and computational modeling on multiple scales. Finally, we provide a perspective on how multi-scale computational models, which couple perfusion changes to hepatic function, could become part of clinical workflows to predict and optimize patient outcome after complex liver surgery.

## 1. Introduction

Liver resection, i.e., removal of part of the liver, is the most important procedure in liver surgery. In Germany, more than 20,000 liver resections are performed annually (Filmann et al., 2019). Due to demographic changes, the incidence of primary and secondary liver tumors increases as patients age. In parallel, the risk of liver surgery increases due to age-associated preexisting liver disease and other comorbidities that affect blood flow to the liver, such as cardiovascular disease.

Extended liver resection remains a high risk procedure, as potential postoperative hepatic dysfunction and eventual liver failure can lead to patient morbidity and even mortality. Removal of large parts of the liver not only poses a high regenerative challenge but also imposes a high metabolic load on the liver remnant (Ray et al., 2018). First, the loss of liver mass impairs the function of the remnant liver through portal hypertension (increase of pressure in the portal venous system) and hyperperfusion (increased perfusion). Both are unavoidable consequences of removing not only hepatic parenchyma but also the vascular bed. Second, extended liver resection compromises hepatic perfusion because of the mismatch between the two supplying portal veins and three draining hepatic veins. Transection of hepatic parenchyma inevitably leads to an impairment of either supply or drainage in the corresponding hepatic region. In addition, the surgical procedure itself carries functional risks (e.g., ischemia-reperfusion injury).

Current preoperative diagnostics allows a detailed anatomical and functional assessment of the liver. As part of the clinical routine, the location of the tumor to be resected is visualized in the context of the patient's vascular anatomy. In case of extended resection, hepatic hemodynamics, consisting of measurement of portal venous flow and pressure is assessed additionally. Furthermore, selected metabolic functions of the liver indicative of the overall function of the liver (e.g., LiMAx or indocyanine green ICG clearance) are usually quantified.

However, current preoperative diagnostics have distinct limitations. Despite high-quality imaging, precise determination of hepatic hemodynamics and sophisticated functional assays, the spatial resolution of specific hepatic functions is still rather low. Although it is known that liver perfusion and function are closely related (Takahashi et al., 2014), it is currently not possible to quantify this relationship, neither for the whole liver nor for a defined liver lobe. Changes in blood flow affect transport to and from regions of the liver (macroscale), in turn changing gradients of oxygen and nutrients in the lobulus and sinusoid (micro-scale), and thus directly impacting metabolic functions. Furthermore, changes in blood flow impose traction, tension and shear forces on liver tissue. Metabolic consequences of those mechanical forces cannot yet be determined, because the molecular links between perfusion and function are unknown. Although perfusion changes are likely sensed via mechanosensors that transmit mechanical forces into the cell, the link to hepatic metabolism is largely elusive.

The liver is the only parenchymal organ capable of near complete regeneration in response to tissue loss. Loss of liver mass by liver resection initiates liver regeneration and tissue remodeling, both necessary to restore tissue homeostasis and volume. Although the physiology and molecular mechanisms involved in liver regeneration have been studied for many years, prediction of the course and outcome of liver regeneration for individual patients is still not possible. The perfusion-associated mechanical forces are crucial for tissue regeneration and remodeling. Both, regeneration and remodeling, are ultimate prerequisites for restoring tissue homeostasis after partial hepatectomy. The molecular basis of functional changes after liver surgery is not well understood because of complex feedback loops, non-linearities, spatial heterogeneities, and different time-scales of events. This complexity requires novel approaches to relate surgically induced alterations in liver perfusion to hepatic metabolic functions. A better understanding of perfusion-function relationships hence is needed to improve preoperative diagnostic and risk assessment. This would allow us to identify patients who benefit most from surgery and those at increased risk for complications.

Systems medicine using multi-scale computational modeling is a unique approach to gain a better understanding of complex biomedical systems, such as the perfusion-function relationship after hepatectomy.

To improve patient-specific risk assessment in the context of liver surgery, computational modeling aims to (i) integrate heterogeneous data and knowledge at multiple scales about how perfusion connects to hepatic function, (ii) generate hypotheses based on integrated models (which need to be validated experimentally and related to clinical data), (iii) support surgical planning by predicting surgically induced perfusion perturbations and their functional (metabolic) consequences, and (iv) minimize surgical risk for the patient.

In this review, we will delineate the relationships between alterations in hepatic perfusion and their consequences for hepatic functions in the context of liver surgery, using hepatectomy as an example. First, we provide an overview of the current knowledge and available tools in clinical and experimental settings. Second, we will discuss how computational models and systems medicine approaches can contribute to a better understanding of the complex perfusion-function interactions. We end with a perspective on how such a systems medicine approach based on multiscale predictive models can be incorporated into the clinical decision-making process.

## 2. Clinical and Experimental Part

### 2.1. Clinical Problem

The term liver resection does not refer to a single surgical procedure, but comprises a wide spectrum of procedures that differ in their respective surgical strategy and technique. Two key surgical strategies are currently in use: Conventional (single-stage) hepatectomies and, for critical extended liver resections, multiple two-stage procedures.

Conventional liver resections involve the removal of one or more anatomically defined liver segments, defined as the hepatic territory supplied by the corresponding portal venous branch. Removal of liver segments requires transection of the hepatic parenchyma. Surgical techniques have been developed to minimize the tissue and vascular damage associated with transection in order to preserve the viability and perfusion of the adjacent liver tissue.

Two-stage hepatectomy is performed when the volume and expected function of the future liver remnant is considered too small to maintain vital metabolic functions for the patient. In the first step, the portal vein branches of the tumor-bearing liver lobe are occluded. Occlusion causes atrophy of the corresponding liver lobe. To compensate for this reduction in functional liver tissue, the volume of the non-ligated liver lobule increases substantially. Once compensatory hypertrophy of the future remnant liver is deemed sufficient to maintain the life-saving functions, the atrophied tumor bearing lobe is resected during the second step.

However, frequently the liver does not regenerate sufficiently because preexisting liver conditions such as steatosis, fibrosis or cholestasis impair the course of regeneration. Furthermore, simple portal vein occlusion without parenchymal transection often leads to a compensatory flow redistribution via existing porto-portal shunts, which reduces the efficacy of this strategy (Deal et al., 2018). To prevent collateral formation, a novel procedure called associating liver partition and portal vein ligation for staged hepatectomy (ALPPS) has been developed (Schnitzbauer et al., 2012). Here, portal vein occlusion is combined with transection of the hepatic parenchyma in the first step, followed by removal of the already mobilized and transected portally deprived liver lobe in the second step. However, in two-stage hepatectomy, the patient must undergo two major operations within a short time period of 7–10 days. Therefore, the indication for this complex procedure is taken with even greater caution.

### 2.2. Preoperative Diagnostics

Currently, there is no generally accepted standard for preoperative diagnostics prior to partial liver resection regarding liver anatomy, technical operability, liver volume and function.

#### 2.2.1. Liver Anatomy, Technical Aspects and Volume Assessment

The minimum requirements are defined in national guidelines. For Germany, the S3 guideline recommends ultrasound of the liver and multiphase contrast-enhanced computed tomography to assess technical operability and to evaluate the expected remnant liver volume and overall parenchymal quality. If there is doubt about the technical operability, more detailed imaging such as additional MRI with liver-specific contrast agent (Barth et al., 2016; Geisel et al., 2017; Wang et al., 2021b) is recommended. However, all contrast-enhanced techniques (CT, MRI and US) represent volume-based procedures and are limited in their predictive power of postoperative organ function.

#### 2.2.2. Liver Function Assessment

In daily clinical routine, most centers rely on standard laboratory parameters covering different aspects of hepatic function to assess overall liver function. Liver enzyme release is taken as an indicator of hepatocellular injury, bilirubin as a marker of excretory function, and serum ChE, albumin and clotting factors as parameters of hepatic protein synthesis. However, this approach has some pitfalls. Although these parameters indicate the condition and main functions of the liver (injury, detoxification, protein synthesis), none of them is considered a reliable marker to quantify either functional hepatic reserve or liver dysfunction in critically ill patients (Nista et al., 2004; Bonfrate et al., 2015). Furthermore, these parameters provide only a static snapshot of liver function.

Currently, additional liver function tests are used in selected hepatobiliary centers prior to complex resections: global liver function assays such as ICG-clearance and the LiMAx-Assay as well as spatially resolved imaging technologies such as scintigraphy with radiolabeled tracers (e.g., mebrofenin-scintigraphy) and contrast-enhanced MRI. All four provide more detailed insight into liver function (metabolism and/or excretion) by reflecting the dynamic elimination of the test substance from the body.

ICG based liver function testing such as ICG-PDR and ICG-R15 is an established clinical tool for the assessment of liver function and perfusion. It is the most commonly used dynamic liver function test performed at bedside. After intravenous injection, ICG is selectively taken up by hepatocytes and excreted into bile. The test is performed using transcutaneous pulse-densitometry, a non-invasive fingertip method, and provides results within 6–8 min. ICG kinetics can be a reliable indicator in the context of liver surgery. ICG-clearance successfully predicted postoperative mortality in cirrhotic patients undergoing hepatic resection unlike other parameters (Hemming et al., 1992) and is a very good prognostic marker for liver failure after hepatectomy (Nonami et al., 1999). Preoperatively impaired ICG results are significantly associated with postoperative liver dysfunction and may predict poor outcome on postoperative day 1 (Haegele et al., 2016).

The LiMAx test is based on the indirect determination of CYP 1A2 activity in hepatocytes. After i.v. injection of ^13^C-methacetin, the CYP1A2 system metabolizes the substance into paracetamol and ^13^CO_2_. Using the spectral laser technique, the ratio of ^13^CO_2_/^12^CO_2_ can be determined via a breath test. The kinetics of ^13^CO_2_ appearance in the expired air thereby indicates the relative liver function (Rubin et al., 2017). The LiMAx test has been applied to predict postoperative outcome after hepatectomy (Stockmann et al., 2009). Post-hepatectomy liver failure and related mortality could be reduced after implementation of a preoperative LiMAx-based patient selection algorithm (Jara et al., 2015). Furthermore, LiMAx has been applied to follow restoration of functional capacity after partial liver resection (Lock et al., 2012; Bednarsch et al., 2016). The prediction of future liver remnant function via LiMAx highly correlated with future liver volume, and can thus be used to estimate postoperative morbidity (Blüthner et al., 2020).

Nuclear medicine scintigraphy imaging techniques exploit the specific properties of different tracers. HBS allows visualization of the specific HEF (Gupta et al., 2018). For this purpose, Technetium (99mTc) mebrofenin is administered intravenously before liver scintigraphy is performed. Mebrofenin is transported into hepatocytes via specific transporter proteins (OATP1B1 and OATP1B3) (Ghibellini et al., 2008) and excreted into the bile canaliculi by MDRP2 (Hendrikse et al., 2004). The areas in the liver where 99mTc mebrofenin accumulates (high uptake) is indicative of regional and global function. There is also potential to quantitatively assess liver function before and after surgical intervention (Rassam et al., 2019b; Uz et al., 2019) such as HEF, mebrofenin uptake rate or hepatic extraction rate (Gupta et al., 2018; Rassam et al., 2019a). The advantage of this method compared to ICG and LiMAx is the spatial resolution, albeit very coarse. Mebrofenin HBS has shown a strong correlation with 15 min ICG clearance (Erdogan et al., 2004). Mebrofenin HBS has been applied to evaluate liver function in hepatectomy (Bennink et al., 2004; Dinant et al., 2007; de Graaf et al., 2010) and showed a strong correlation between preoperative remnant liver function and the actual 1-day post-hepatectomy measurement (Bennink et al., 2004).

Other functional tracer-based imaging technologies used to assess liver function include SPECT and PET. SPECT is a nuclear imaging scanning methodology that integrates CT and a radioactive tracer such as sulfur colloid. Uptake of the tracer by the liver is an indicator of hepatic function. PET also estimates liver function based on the uptake and clearance of different radioactive positron-emitting tracers (e.g., ^18^F FDGal) (Bak-Fredslund et al., 2017; Keiding et al., 2018) and has been applied to predict postoperative liver function (Cho et al., 2017). Multi compartment models are employed to derive further tissue parameters, like hepatic arterial or portal blood flow, hepatic arterial or portal perfusion index or blood volume, from the dynamic time course of the contrast agent passing through the tissue (Wang et al., 2021a).

MRI is a non-ionizing imaging technique routinely used to detect hepatic tumors (Liu et al., 2017). More detailed analysis of the time course of the liver-specific contrast agent Gd-EOB-DTPA also allows to assess liver function by imaging its spatially resolved uptake and excretion into the bile by the hepatocytes (Wang et al., 2021b). Dynamic Gd-EOB-DTPA imaging has been applied to evaluate preoperative remnant liver function and post-hepatectomy outcome (Yoon et al., 2016; Itoh et al., 2017; Asenbaum et al., 2018; Chuang et al., 2018; Kim et al., 2018; Araki et al., 2020; Wang et al., 2021b).

### 2.3. Surrogate Approaches to Assess Liver Function

#### 2.3.1. Assessment of Liver Stiffness

Liver diseases not only affect hepatic function, but also lead to morphological changes, which in turn alter the mechanical properties of the tissue. Most diseases lead to increased stiffness of the tissue, e.g., liver fibrosis results in enhanced stiffness due to an increased ECM (Wells, 2005; Li et al., 2020b). Recently, hepatic elastography has gained attention, a medical imaging modality that relies on sound waves or forced tissue vibrations to measure tissue elastic properties and stiffness. It can be performed in combination with US or MRI. Correlations exist between liver elasticity and liver functional reserve, as demonstrated with ICG (Sugiura et al., 2019) or LiMAx (Heucke et al., 2019).

Clinically, a variety of US elastography methods have been developed. SWE and ARFI are the dominant methods in clinics today, offering integration with other advanced US imaging modalities (Ferraioli, 2019).

Alternatively, by using an external vibration generator, elastography can be performed with MRI. Current literature generally attributes higher diagnostic performance and fewer technical failures to MRI compared to US methods (Yin and Venkatesh, 2018). MRE also offers great potential for further development of new multiparametric methods to distinguish processes like inflammation, fibrosis, venous congestion and portal hypertension (Roldán-Alzate et al., 2015; Palaniyappan et al., 2016; Frydrychowicz et al., 2017; Yin et al., 2017; Leung et al., 2018), and has been successfully used to predict outcome after hepatectomy (Lee et al., 2017; Sato et al., 2018) and regeneration capacity (Jang et al., 2017). In the evaluation of NAFLD, MRI has the added advantage of providing an independent method for fat quantification (Zhang et al., 2018).

#### 2.3.2. Quantification of Intrahepatic Fat

A clinically frequently observed pathological liver condition that affects liver perfusion, function, and recovery is hepatic steatosis. Hepatic steatosis assessed by routine preoperative MRI has been shown to be an independent risk factor of severe postoperative complications after major liver resection (d'Assignies et al., 2016).

While US, CT, and MRI can be used to assess hepatic steatosis *in vivo*, PDFF determination with MRI is currently the most accurate imaging method for quantification (Zhang et al., 2018; Troelstra et al., 2021).

#### 2.3.3. Assessment of Hemodynamics and Perfusion

Preoperative assessment of hemodynamics and perfusion relies mainly on noninvasive technologies, whereas intraoperative assessment is also performed with direct invasive techniques. The two main noninvasive technologies, US and MRI, can quantify blood flow in the major supplying and draining vessels of the liver (Yzet et al., 2010; Chouhan et al., 2017). Doppler US typically provides localized, dynamic flow measurements.

Similar to water-fat quantification, the evaluation and quantification of tissue perfusion with MRI has a long history (Rinck et al., 1984; Rosen et al., 1990). Perfusion is defined here as blood delivery at the capillary level. Over the years, two main approaches to perfusion MRI have been developed. The first uses an exogenous contrast agent (Jahng et al., 2014) and includes DSC-MRI and DCE-MRI (Leporq et al., 2018; Weiss et al., 2019). DCE-MRI techniques allow quantitative characterization of parenchymal and (lesion) microcirculatory changes (Thng et al., 2010) and the study of liver damage (Byk et al., 2016; Lu et al., 2017). The assessment and derivation of the tissue parameters, like the perfusion and uptake of contrast agent as correlate of liver function, is facilitated by multi compartment models fitted to the dynamic time course of the MRI data (Simeth et al., 2018).

The second approach refers to ASL (Williams et al., 1992), in which magnetically labeled blood itself is used as an endogenous tracer and its tissue accumulation is measured (Johnson et al., 2016). By using different, carefully placed labeling planes, arterial and portal perfusion can be assessed separately (Martirosian et al., 2019).

Invasive assessment of hepatic hemodynamics involves direct measurement of portal and hepatic arterial flow rates using US Doppler technology. Determination of portal pressure requires placement of a pressure sensor in the vessel of interest. Another valuable parameter is the HVPG, which is usually measured by inserting a balloon catheter into a branch of the hepatic vein via the jugular vein. HVPG has been applied in the context of hepatectomy showing an association of outcome with preoperative HVPG and a cutoff of HVPG < 10 mmHg was proposed (Boleslawski et al., 2012; Cucchetti et al., 2016). Although progress has been made in the noninvasive assessment of portal hypertension (Gouya et al., 2016) it remains a clinical challenge (Wan et al., 2021).

#### 2.3.4. Assessment of Tissue Density by DWI

DWI is an MRI technique that is sensitive to the mobility of water molecules in tissue and therefore can provide insight into local tissue changes. Additionally to the actual self-diffusion of water, the perfusion of tissue causes IVIM in the capillary system of the tissue. These two diffusion effects can be separated with DWI measurement using multiple, differently weighted DWI scans (Mürtz et al., 2018; Fujimoto et al., 2021). Sheng et al. demonstrated that DWI can detect and distinguish microstructural tissue changes during ALPPS and PVL procedures (Sheng et al., 2018). DWI has been applied to assess hepatic ischemia and reperfusion injury (Lu et al., 2017; Yang et al., 2021) and to predict survival after partial hepatectomy (Muhi et al., 2013).

In summary, the selection of a particular procedure for an individual patient is based on the results of the extensive preoperative assessment. The diagnostic strategy in preoperative assessment is tailored to the needs of the patient and follows the standards of the particular center. Current approaches provide a detailed, spatially resolved, albeit indirectly measured assessment of liver function. However, the improved imaging modalities are standing alone and individual tissue parameters are difficult to interpret. The integration of different tissue parameters in descriptive models has been realized (Wang et al., 2016) but currently spatially resolved tissue parameters are not incorporated into complex multiscale predictive models of the liver.

### 2.4. Experimental Approaches in Animal Models

#### 2.4.1. Historical Overview

**Figure 1:**
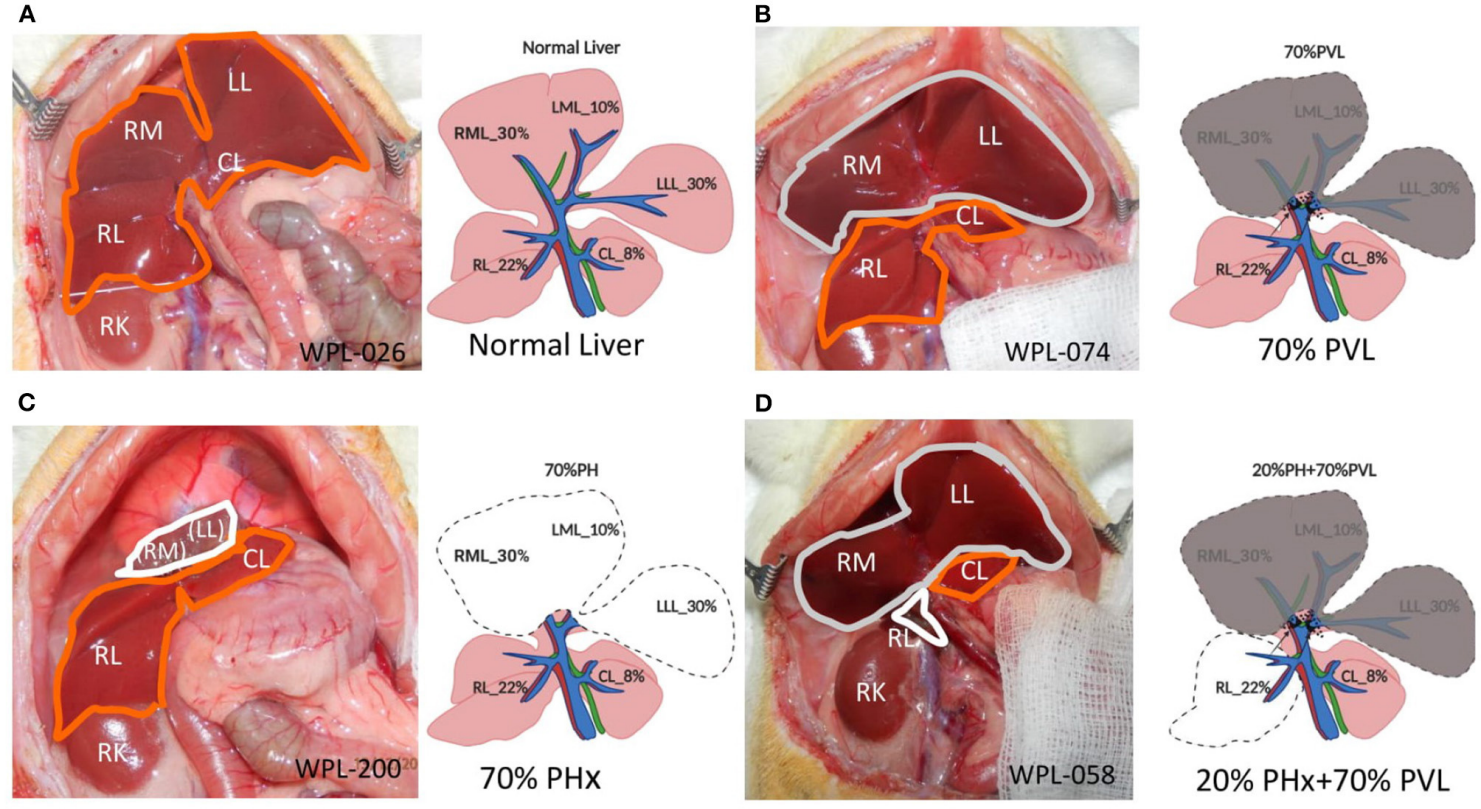
Surgical procedures. **(A)** Open situs with liver (encircled in orange). **(B)** 70% portal vein ligation. Note the slightly darker color of the ligated median and left lateral lobe (encircled in gray) compared to the fresh color of the right and upper caudate lobe (encircled in orange) **(C)** 70% partial hepatectomy. Note the stump (encircled in white) above the right lobes (encircled in orange). **(D)** Combined 20% partial hepatectomy with 70% portal vein ligation: Note the dark color of the portally ligated median and left lateral lobe (encircled in gray), the fresh red color of the upper caudate lobe (encircled in orange) and the stumps from the right lobes (encircled in white). LLL left lateral lobe, ML median lobe, RL right lobe, Cl upper caudate lobe, RK right kidney, LLL, ML, and RL stumps of the respective lobe.

A main limitation for clinical research is the availability of tissue-based data. Human liver tissues can be obtained during surgical procedures and by liver biopsy. For ethical reasons, patients cannot be subjected to repeated liver biopsies pre- and postoperatively. Therefore, animal experiments are important to better understand the pathophysiological mechanisms and processes governing liver surgery and liver regeneration (see Figure 1).

Experimental liver resection in small animals was first performed by Higgins (1931). Originally, 70% of the liver mass was removed after mass ligation of the wide stump of the median and left lateral lobe of the rodent liver, resulting in impaired hepatic outflow and congestion of the remnant liver. With the refinement of surgical techniques (see Table 1), the parenchyma-preserving vessel-oriented technique was established (Madrahimov et al., 2006). Avoidance of congestion and necrosis of the stumps allowed rat survival even after extended 90% resection, which is lethal when using the mass ligation technique. In contrast, additional ligation of the portal vessel reduces the functional remnant liver mass and prevents survival after 90% PHx.

**Table 1:** Overview of different liver resection techniques, differing in how the vascular structures (portal triad and hepatic vein) are ligated respectively transected.

**Reference**	**Species**	**Incision**	**Extent of resection**	**Resected liver lobule**	**Ligation of portal triad**	**Ligation of hepatic vein**
**Liver resection**				
Higgins (1931)	Mouse	Midline	70% PHx	LL, ML	Not done	Parenchymal mass ligation
Weinbren and Woodward (1964)	Rat	Midline	90% PHx	LL, ML, RSL, CL	Not done	Mass ligation
Gaub and Iversen (1984)	Rat	Midline	90% PHx	LL, ML, RSL, RIL	Not done	Parenchymal mass ligation Mass ligation
Kubota (1997)	Rat	Midline	90% PHx	LL, ML, RSL, RIL	Ligation of PV and HA	Piercing ligation
Madrahimov et al. (2006)	Rat	Horizontal laparotomy	90% PHx	LL, Ml, RSL, RIL	None	Clamping and piercing suture
Aller et al. (2009)	Rat	Midline	90% PHx	LL, ML, RSL, RIL	Ligation of PV and HA	Selective ligation of HV

*LL, left lobe; ML, median lobe; RL, right lobe; CL, caudate lobe; RSL, Right supperior Lobe; RIL, Right inferior lobe*.

A bit earlier, in 1920, the first experimental PVL was performed in rabbits by Rous and Larimore (1920). Comparative studies revealed that the time course of liver regeneration after partial resection or PVL followed different kinetics. After simple hepatectomy, hepatocyte proliferation peaks on day 1 in rats and on day 2 in mice (see Table 1) and declines rapidly thereafter. Within a week, the original liver mass is restored.

In both models, the regenerating liver lobes are hyperperfused. However, in the case of PVL, the regenerative requirement is initially much lower. Resection results in immediate loss of function because a substantial amount of liver tissue is removed. In contrast, PVL only compromises function, as the portally deprived lobe is still perfused with arterial blood and thus can contribute to the overall liver function. Therefore, hepatocyte proliferation after PVL in rats peaks later, on postoperative day 2, but lasts for several days (Rozga et al., 1986; García-Pérez et al., 2015). Along with the development of hepatic atrophy, the regenerative need increases, resulting in a reduced but prolonged regenerative response (see Table 2).

**Table 2:** Selection of different rodent models of liver resection/partial hepatectomy (PHx).

**Reference**	**Species**	**Operation**	**Surgical technique**
**Sequential procedures**			
Saito et al. (2006)	Rat	Repeat 70% PHx	Impaired regeneration after 2^nd^ 70% PHx
Sugimoto et al. (2009)	Rat	2-stage PVL^**^	Two stage PVL more effective than single stage PVL
Li et al. (2015)	Rat	BDL followed by + 70% PHx	Impairment of regeneration after BDL
García-Pérez et al. (2015)	Rat	70% PVL + ALPPS	Combined PVL+ ALPPS more effective than PVL alone
Wei et al. (2016)	Rat	70% PVL + ALPPS + 10% PHx	Combined 70% PVL, median lobe transection + caudate lobe resection enhances regeneration of future liver remnant compared to PVL only
**Simultaneous procedures**			
Dirsch et al. (2008a)	Rat	50% PHx + RMHV ligation	Recovery from pericentral necrosis in outflow obstructed liver by induction of pericentral hepatocyte proliferation
Huang et al. (2014)	Rat	70% PHx + RMHV ligation	Impaired recovery from focal outflow obstruction due to reduced hepatic arterial flow through inhibition of nitric oxide production
Ren et al. (2015)	Rat	90% PVL + BDL	Faster induction of atrophy/hypertrophy complex by simultaneous bile duct and portal vein ligation compared to PVL alone
Kawaguchi et al. (2019)	Rat	90% PVL + 30% LLHVL	Regeneration of FLR promoted by additional LLHVL
Wei et al. (2020)	Rat	20% PVL + 70% PHx, 70% PVL + 20% PHx	Induction of regeneration in portally deprived liver lobes by additional resection

*PHx, partial hepatiectomy; PVL, portal vein ligation; ALLPS, associating liver partition with portal vein ligation for staged hepatectomy; RMHVL, right median hepatic vein ligation; LLHVL, Left lateral hepatic vein ligation; BDL, bile duct ligation*.

** *2-stage PVL: 70% PVL (ML+LLL) as first stage, after 7 days, 20% PVL (RL) as second stage*.

Several combined procedures were introduced to better understand regulation of liver regeneration. Sequential procedures include repeated hepatectomy and 2-stage PVL to elucidate the proliferative capacity of the regenerating liver (Saito et al., 2006; Sugimoto et al., 2009). The impact of obstructive jaundice on liver regeneration was studied by first performing bile duct ligation 1 week prior to liver resection (Li 2014). Different models of 2-stage hepatectomy (e.g., 70% PVL with (ALPPS) or without transection of the median lobe followed by PHx of atrophied liver lobes) were developed to better assess the impact of preventing collateral formation (García-Pérez et al., 2015; Wei et al., 2020). Here actually, the development of animal model only happened after introducing the procedure into clinic (Schnitzbauer et al., 2012).

Liver resections and portal occlusions have also been combined with other interventions to better understand factors affecting hepatocyte proliferation and liver regeneration. These include interventions affecting hepatic perfusion such as right median hepatic vein ligation (Dirsch et al., 2008a; Huang et al., 2014). To better understand the impact of additional damage to the portally ligated lobe, bile duct ligation was performed resulting in increased regeneration of the FLR. A similar effect on regeneration of the FLR was observed when inducing congestion of the portally ligated lobe by performing an additional ligation of the left lateral hepatic vein along with PVL. Combination of an additional resection with PVL induced hepatocyte proliferation in the portally deprived liver lobe (Wei et al., 2020). In conclusion, the wide spectrum of surgical models, which emerged over the years, is very useful to investigate the different facets of liver regeneration and the underlying complex pathophysiological mechanism (see Table 2).

#### 2.4.2. Regulatory Molecular Networks in Regeneration

In the past, animal models of liver resection were mainly used to study the molecular mechanism underlying the course of liver regeneration (see Table 3). Many experimental studies have focused on investigating single molecular pathways governing central processes involved in regeneration, such as proliferation, inflammation, angiogenesis (vessel formation process), and recently also autophagy (cell survival process), through classical interventional studies. Specific blocking and reintroduction of selected molecules was performed to elucidate their relevance in liver regeneration.

**Table 3:** Selection of classical interventional studies exploring molecular pathways.

**Reference**	**Species**	**Model**	**Targeted process**	**Target**	**Pharmacological intervention**	**Mechanism**
**Proliferation**						
Adas et al. (2016)	Rat	70% PHx	Proliferation	Stem cell	MSC + VEGF	Injection of MSCs and VEGF-transfected MSCs into portal vein following liver resection increased bile duct and liver hepatocyte proliferation
Ren et al. (2008)	Rat	70% PHx	Angiogenesis	Oxygenation	Hyperbaric oxygen	Angiogenesis effect of HBO-PC on liver after partial hepatectomy is possibly due to increased HIF-1alpha activity and VEGF expression
Yoshida et al. (2011)	Rat	70% PHx	Angiogensis	VEGF, eNOS	L-NAME	Endothelial NOS and VEGF coordinately regulate SEC proliferation during liver regeneration.
Moreno-Carranza et al. (2013)	Rats	PRL-KO-mice	Angiogenesis	Pro-angiogenic hormones	Prolactin	PRL stimulates liver regeneration by upregulation of angiogenesis.
von Heesen et al. (2015)	Rat	70% PHx	Inflammation and angiogenesis	TGF, VEGF	Cilostazol	Proliferation is promoted via inhibiting TGF-β and up-regulating VEGF
Jepsen et al. (2015)	Rat	70% PHx	Inflammation	Cortison	Dexamathasone	Low dose dexamethasone targeted to Kupffer cells does not affect histological liver cell regeneration after 70% hepatectomy in rats, but reduces the inflammatory response judged by circulating markers of inflammation.
Lin et al. (2015)	Mouse	70% PHx	Autophagy	mTOR	Amiodarone Chloroquine	Regeneration is promoted via activating autophagy, respectively impaired by inhibition
Lu et al. (2018)	Mouse	PHx	Autophagy	miRNA	miR-1907	Regeneration is promoted via activating autophagy

*Targeted processes: proliferation, angiogenesis, inflammation, autophagy. MSC, mesenchymal stromal cell; TGF, transforming growth factor; VEGF, vascular endothelial growth factor; HIF-1α, hypoxia-inducible factor-1α; eNOS, endothelial nitric oxide synthase*.

#### 2.4.3. Interaction Between Regeneration and Metabolism

Metabolic pathways regulating energy homeostasis are central to liver regeneration. Their role has been studied in liver resection, particularly in knockout models (see Table 4). For example, lack of sirtuin and PPARβ reduced energy metabolism and inhibited regeneration (Liu et al., 2013, 2019). In contrast, lack of PTEN and aldolase reductase increased energy metabolism and induced regeneration (Kachaylo et al., 2017; Li et al., 2020a).

**Table 4:** Interaction between resection–regeneration and metabolism.

**Reference**	**Species**	**Model**	**Treatment**	**Metabolism change**		**Regeneration**
				**Energy**	**Biomarker**	
**Inhibition of liver regeneration**						
Gutiérrez-Salinas et al. (1999)	Rat	70% PHx	ethanol	ATP↓	Redox-Pair↓	Liver wet weight↓
Picard et al. (2004)	Rat	70% PVL	Retrorsine		Caspase 3 ↑, TNF-α ↑	Proliferation inhibition and apoptosis activation
Liu et al. (2013)	PPARβKO Mouse^a^	70% PHx	PPARβknockout	-	Hexokinase 2^b^↓	Ki67↓, Cyclins ↓
Liu et al. (2019)	Sirt6LKO mouse^c^	70% PHx	Sirtuin 6 knockout	-	Glucose↓	Ki67↓, Cyclins↓
**Induction of liver regeneration**						
Liska et al. (2009)	Pig	50% 60% PVL	MSC			Liver regeneration stimulation
Damrauer et al. (2011)	Mouse	78% PHx^d^	A20^e^	-	PPAR α^f^↑	Ki67 ↑
Kachaylo et al. (2017)	PtenKO Mouse	68% PHx	PTEN^g^ knockout		Glycogen ↑, TGs↑	Liver wet weight ↑
Tautenhahn et al. (2016)	Rat	extended liver resection	MSC	Increased lipid oxidation	Impairment of mitochondrial oxidation after resection	Ki67↑, hepatocyte apoptosis ↓
Li et al. (2018b)	Mouse with fatty liver	40% PHx during 60% WIR^h^	ApoA-1	-	PGC-1α ↑	PCNA↑, Cyclins↑
Li et al. (2020a)	ARKO Mouse^i^	40% PHx during 60% WIR	Aldose reductase knockout	ATP ↑	AMPK ↑	Cyclins ↑
Cheng et al. (2018)	Mouse	70%	PHx	PPARγ2	HGF-cMet-ERK1/2 HGF	Hepatocyte proliferation abrogation via inhibiting HGF-cMet-ERK1/2 pathway

*MSC, mesenchymal stromal cells; PPARγ, peroxisome proliferator-activated receptor-gamma; HGF, hepatocyte growth factor*.

a *Peroxisome proliferator-activated receptor β knockout mouse, PPARβ regulates energy homeostasis and cell proliferation*.

b *Related to glycolysis*.

c *Sirtuin 6 knockout mouse, Sirtuin 6 is an NAD+-dependent deacetylase*.

d *Resection of lateral, medial, left and right lobes*.

e *NF-kB inhibitory protein*.

f *Peroxisome proliferator-activated receptor α, which promotes mitochondrial ATP*.

g *Phosphatase and tensin homolog (PTEN) knockout mouse. PTEN is an inhibitor of AKT / mTOR axis*.

h *Clamping the branch of hepatic artery and portal vein to the right and triangle lobes for 45min and hepatectomy of left and caudate lobes performed during the ischemia duration*.

i *Aldose reductase (AR) knockout mouse*.

#### 2.4.4. Hepatic Hemodynamics and Microcirculation

Few studies addressed the investigation of hepatic hemodynamics and microcirculation in small animals subjected to various hepatobiliary procedures (see Table 5). They are mainly descriptive in nature. This may be partly due to the technical difficulties in assessing hepatic hemodynamics and microcirculation in such models. Hepatic hemodynamics can be assessed in rats using standard equipment (fluid filled catheters and ultrasound flow probes). The same procedure is also feasible in mice (Xie et al., 2016), but is more challenging due to their small size. Both portal pressure and portal venous blood flow per liver weight in small animals are comparable to that in humans. Resection or portal vein occlusion induce portal hypertension, hepatic hyperperfusion, and in humans, FOO. Some experimental studies focused on describing and modulating resection-induced impairment of hepatic hemodynamics. Fewer studies aimed for interfering by surgical or pharmacological interventions (e.g., splenectomy, drug treatment). Good examples are the reports by Huang et al. (2014) and Arlt et al. (2017), both of whom used a rat model of 70% PHx combined with right median hepatic vein ligation. This combined procedure mimics resection-associated FOO due to hepatic vein transection. Dahmen's group explored the impact of different interventions (e.g., application of vasoactive drugs like Molsidomine, L-NAME) on the formation of sinusoidal vascular canals during the spontaneous recovery process from FOO (Arlt et al., 2017).

**Table 5:** Rodent (liver resection) models impacting on hepatic perfusion (in the context of liver surgery).

**Reference**	**Species**	**Surgical model**	**Surgical technique**	**Treatment and result**	**Peak of Proliferation (POD)**	**Alterations in hepatic perfusion**
Hossain et al. (2003)	Rat with cirrhotic liver	15 min WIR and 50% PHx^a^	Liver mass ligation and removal	PGE^b^	-	Increase of portal venous flow
**Impact of resection on hepatic hemodynamics**						
Dahmen et al. (2007)	Rat	stepwise resection (30%, 70%, 90% & 95%)	Piercing sutures for PH	No drug	nd	Stepwise liver resection causes non-linear increase of portal venous flow and pressure
Xie et al. (2016)	Mouse	70% PHx	Piercing sutures for PH	No drug	POD2	70% PHx causes increase of portal venous flow and pressure
Eipel et al. (2010)	Rat	PHx of 30%,70%,85% and 90%	Liver mass ligation + removal of spleen	No drug	nd	Splenectomy leads to decrease of portal hyperperfusion and increased arterial blood supply and liver regeneration
Zhuang et al. (2012)	Rabbit	80% PHx	Not indicated	Splenectomy	nd	Splenectomy decreased resection-induced portal vein pressure
Tautenhahn et al. (2017)	Pig	70% PHx	Pringle maneuver	MSC		MSC correct hemodynamic dysfunction after liver resection
**Impact of focal outflow obstruction on regional hepatic perfusion and microcirculation**						
Dirsch et al. (2008b)	Rat	50% PHx (LLL; RSL; RIL; CIL; SL) + RMHVL	Piercing sutures for PH	No drug	POD2	Spontaneous recovery from FOO is associated by formation of sinusoidal vascular canals
Huang et al. (2011)	Rat	Syngeneic Ltx + RMHVL	Piercing sutures for PH	No drug	nd	Spontaneous recovery from FOO and formation of sinusoidal vascular canals is dependent on arterial blood supply
Huang et al. (2014)	Rat	70% PHx (LML; LLL; RSL; RIL; CIL; SL) + RMHVL	Piercing sutures for PH	Molsidomin, L-NAME	POD1	Spontaneous recovery from FOO and formation of sinusoidal vascular canals is impaired by L-NAME
Arlt et al. (2017)	Lew-Rat	70% PHx (LML; LLL; RSL; RIL; CIL; SL) + RMHVL^c^	Piercing sutures for PH	Splenectomy, Carvedilol, ISMN^d^ Sildenafil, Octreotid,	POD1	Drugs cause slight reduction of portal pressure and slight increase of HAF, but have no impact on PVF and hepatic damage
Englert et al. (2019)	Rat	Isolated liver		Hemin infusion 10 min		Perfusion pressure of isolated liver increase
Iwasaki et al. (2019)	Rat	BDL5 + 50% PHx	Liver mass ligation and removal	L-NAME	-	Microcirculation of the liver with BDL

a *50% PHx was performed during 15 min ischemia (occluding hepatoduodenal ligament)*.

b *Prostaglandin E_1_*.

c *RMHVL: right median hepatic vein ligation*.

d *ISMN: Nitrovasodilator isosorbide-5-mononitrate*.

Two different technologies are commonly used to assess hepatic microcirculation: intravital microscopy with fluorescent dyes and dark-field microscopy. Intravital microscopy is frequently performed in animal experiments. It allows the assessment of fluidic flow based on the injection of fluorescent albumin, but also intravascular labeled blood cells as well as the migration of blood derived cells into the hepatic parenchyma. However, because of the required injection of fluorescently labeled molecules or cells, it is not used clinically. Dark-field microscopy also allows quantification of blood flow velocity (Dahmen et al., 2007) and has occasionally been applied in clinical studies (Puhl et al., 2003).

As early as 1981, Lautt (1981) described a seminal phenomenon, the hepatic arterial buffer response. He was the first to understand that maintenance of hepatic flow is of paramount importance to maintain homeostasis (Lautt, 2007). Since the liver cannot directly control portal venous blood flow, there are a number of interrelated mechanisms to compensate for changes in portal blood flow that occur after PHx. PHx results in decreased portal blood flow, which in turn results in reduced (1) intrahepatic distending pressure. This causes the highly compliant hepatic vasculature to passively expel half of its blood volume, thereby increasing venous return, cardiac output, and HBF. The reduction in portal blood flow also causes (2) activation of the hepatic arterial buffer response and (3) an HBF-dependent hepatorenal reflex (Eipel et al., 2010). Adenosine is constantly released into the Mall space, a small fluid-filled space surrounding the terminal branches of the hepatic arterioles, portal venules and sensory nerves. Adenosine concentration is regulated by washout into the portal venules. In case of reduced portal flow, the washout is also reduced and adenosine may accumulate. This in turn leads to dilatation of the hepatic artery, thereby compensating for the reduced portal flow.

Elevated levels of adenosine activate the surrounding sensory nerves, which in turn triggers a reflex causing fluid retention in the kidney, also increasing the total blood volume, thereby maintaining cardiac output and PF. When hepatic blood flow cannot be maintained, other mechanisms, such as a shear stress/nitric oxide-dependent mechanism, are activated and trigger hepatocyte proliferation or apoptosis to achieve a better match between total liver mass and blood supply (Vollmar and Menger, 2009). These mechanisms are unique to the liver and its vascular bed and demonstrate how a major homeostatic organ is subject to multiple integrative regulatory mechanisms. However, to date, few efforts have been made to understand the molecular mechanism of blood flow-induced mechanical forces underlying the regulation of liver function and regeneration.

In summary, a variety of experimental approaches have been developed to investigate the regulatory processes involved in liver regeneration after partial hepatectomy. However, few studies have been designed for a comprehensive evaluation of the interplay between physical challenges, perfusion changes, their recognition by mechanoperception and the impact on regeneration and metabolism.

### 2.5. Mechanoperception in the Liver and Impact on Cellular Metabolism

#### 2.5.1. Hemodynamic Changes After PHx May Trigger Liver Regeneration

Liver regeneration to restore tissue loss after surgical removal or toxic insult occurs through proliferation of both parenchymal and non-parenchymal cells of the liver. In loss due to PHx, proliferation of hepatic cells is distributed throughout the parenchyma, and the factors that trigger and maintain liver cell proliferation during regeneration have been described (Michalopoulos, 2017). However, the primary trigger sensing the parenchymal loss remains largely elusive.

Hepatectomy markedly changes blood flow in the remnant liver featuring, e.g., portal hypertension and arterial hypoperfusion. Experimental evidence suggests that flow changes may regulate regeneration. For example, in pigs, increases in total hepatic flow after PHx preceded the increase in liver regeneration. Changes in hepatic flow correlated with the extent of liver mass loss, resulting in a 2-3-fold increase in hepatic perfusion and a 10–30% increase in portal pressure, suggesting a quantitative relationship (Kahn et al., 1984; Dahmen et al., 2007).

Surgically induced increase in portal blood flow appears to be critical for regeneration. In dogs, liver regeneration was hampered when a portocaval shunt, reducing portal blood flow, was added to hepatectomy (Mann et al., 1931). Patients with poor clinical outcome experienced a significant decrease in portal flow during extensive hepatectomy, suggesting that an adequate increase in portal blood flow is essential for hepatic regeneration (Kawasaki et al., 1991). Similarly, post-hepatectomy outcome improved in patients featuring higher portal flow postoperatively. Functional improvements like bilirubin levels and hepatic growth rate correlated with mean portal flow velocity (Kin et al., 1994; Hou et al., 2018). In humans, portal blood flow was inhomogeneously distributed in the remnant portal branches after PHx, as was the distribution of hepatocyte proliferation, suggesting a causal relationship between heterogeneous distribution of portal blood flow and regeneration (Iimuro et al., 2013).

Yet, there is some evidence against portal blood flow being the only regulator of liver regeneration. Minor (10–30%) removal of liver mass results in only a marginal regenerative response, suggesting that a threshold change in portal blood flow is required to initiate appropriate compensatory growth (Abshagen et al., 2012). Moreover, liver regeneration may occur even in the absence of portal blood flow. Despite ligation of the portal branch, a moderate proliferative response was observed in the corresponding ligated liver lobe, especially after an additional liver resection (Weinbren, 1955).

Although portal hyperperfusion might not be indispensable, the importance of flow-related mechanical forces has been repeatedly demonstrated. Mechanical inflictions induced by flow changes may play a major role in both the initiation and the termination of liver regeneration (Song et al., 2017). This is corroborated by the inverse quantitative correlations between the increase in portal blood flow and the remnant liver volume, which is accompanied by the increase in hepatic shear stress stimulating liver mass restoration. Likely, the increase of the blood flow-to-liver mass-ratio immediately after PHx and the resulting increased intrahepatic shear stress stimulate and regulate liver regeneration (Niiya et al., 1999; Sato et al., 1999; Schoen et al., 2001; Nobuoka et al., 2006). Conversely, reduction of shear stress in the liver by portocaval shunts resulted in liver atrophy (Sato et al., 1997; Abshagen et al., 2012).

#### 2.5.2. Changes in Hemodynamics May Be Sensed and Trigger Cellular Responses

Changes in blood flow exert forces on liver tissue. Because hepatic sinusoids are likely the first to sense changes in hepatic flow, the sinusoidal endothelium may play a major role in transducing these forces (Shu et al., 2021). This is supported by the hierarchical topology of the hepatic sinusoids involving cell-cell and cell-matrix interactions. Hepatocytes communicate directly with the sinusoids via the ECM. The ECM is connecting the extraluminal side of endothelial cells and the sinusoidal face of hepatocytes, thereby bridging the space of Disse. Indirect communication connects cells in the space of Disse, like HSC via the ECM to endothelial cells, and in turn hepatic stellate cells to hepatocytes. Direct cell-cell contacts between adjacent hepatocytes maintain epithelial hepatocyte-to- hepatocyte communication (Kang, 2020).

Cellular adhesion molecules, which transmit mechanical forces into cells, mediate cellular contacts to the ECM or to neighboring cells. In focal adhesion contacts, integrins connect cells and the ECM and serve as receptors for components of the ECM like fibronectin and collagens. Mechanical challenges of the ECM induce conformational changes in the integrin chains, followed by integrin clustering and intracellular activation of signaling pathways that include activation of, e.g., FAK, phospholipase C, and PI3K and others (Alexius, 1991). Besides transmission of mechanical forces acting on the ECM, integrins transmit intrinsic properties of the ECM to anchored cells. In the healthy liver, quiescent HSC and sinusoidal endothelial cells create a homeostatic ECM of relatively low stiffness in the space of Disse, which is necessary for hepatocyte function linked to normal hepatocyte polarity (Müsch, 2014). In the fibrotic liver, activated stellate cells produce ECM featuring augmented stiffness, which affects hepatocyte polarity and function. TGFβ, the major mediator of liver fibrosis, is activated by release from its latent integrin-associated form, thus responding to any conformational change of the ECM, either triggered by mechanical challenges or by changes of the ECM composition and stiffness (Hintermann and Christen, 2019).

**Figure 2:**
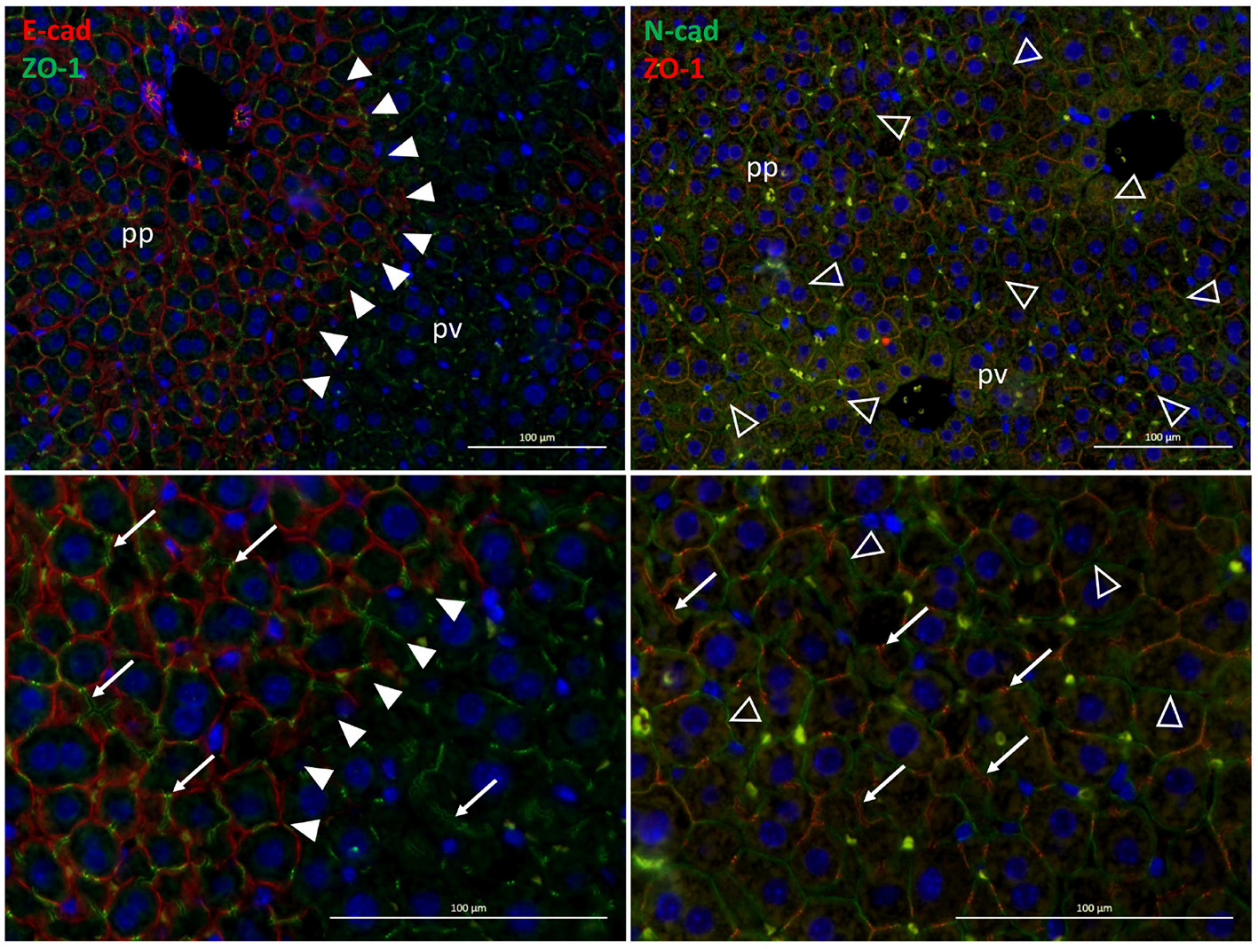
Zonal expression of E-cadherin (red, left panels)and of N-cadherin (green, right panels) in mouse liver sections. The exclusive periportal expression of E-cadherin demarcates the boundary between periportal and perivenous hepatocytes in the liver lobule (solid arrowheads). In contrast, N-cadherin is expressed all over the hepatic parenchyma (open arrowheads). Also, the tight junction protein ZO-1 (green, left panels; red, right panels) designating the bile canaliculi (white arrows) is expressed pan-parenchymally. Lower panels are digital magnifications of images shown in the upper panels. pp, periportal; pv, perivenous.

The major cell adhesion molecules include E- and N-cadherin. In the rodent liver, E-cadherin is expressed in periportal areas, whereas N-cadherin is expressed throughout the parenchyma (see Figure 2). By homodimeric binding of the extracellular domains of cadherins on adjacent cells, they form adherens junctions that link the junction complex to the cytoskeleton by connecting the intracellular domains of the cadherins via p120, β-catenin and α-catenin to actin. Thus, physical forces, as likewise induced by sinusoidal flow changes, affect cellular behavior in terms of proliferation, differentiation and tissue homeostasis (Buckley et al., 2014); (KEGG pathway entry: hsa04520). Further, changes in mechanical forces outside the cell are transmitted into the cell by the tight junction complex comprising occludins, claudins and JAM. These can couple to the actin filament system via interactions with ZO. The tight junction complex can activate intracellular signaling pathways via PKC, PI3K and others that affect cell polarity, differentiation, and paracellular transport (Chiba et al., 2008); (KEGG pathway entry: hsa04530).

Similar to the zonal expression of E-cadherin, metabolic functions are expressed heterogeneously in the hepatic parenchyma. For example periportal hepatocytes surrounding the branches of the portal vein are specialized in producing glucose by gluconeogenesis, whereas perivenous hepatocytes surrounding the branches of the hepatic vein utilize glucose by glycolysis. The zonal pattern of metabolic functions is dynamic and regulated by hormone, substrate and oxygen gradients forming in the blood during passage through the sinusoids (Kietzmann, 2017). These may change by variations in the nutritional state of the organism, by physical activity and in liver diseases with functional consequences for the whole organism (Jungermann and Kietzmann, 2000; Steinman et al., 2021). On the molecular level, hepatic zonation is regulated, besides others, by morphogenic pathways including the Wnt, Hedgehog and YAP/TAZ pathways, which interestingly are involved in both mechanotransduction and metabolic imprinting of the liver (see below) (Hu et al., 2017b; Kolbe et al., 2019; Wild et al., 2020). PHx has a major impact on the metabolic zonation of the hepatic parenchyma. In the rat, the gluconeogenic capacity of the liver is increased by increasing gluconeogenesis, while glycolysis decreases, which abrogates the zonal gradients of these opposing metabolic pathways (Andersen et al., 1984; Chatzipanagiotou et al., 1985). A very obvious sign of changes in hepatic metabolism is the accumulation of triglycerides after PHx. Surgery-induced stress releases fatty acids from adipose tissue that enter the liver to provide energy substrates for liver regeneration (Lafontan et al., 1997; Walldorf et al., 2010). However, in steatohepatitis and, due to the small liver remnant after extended hepatectomies, excess lipid load occurs and impairs post-hepatectomy regeneration, likely as a consequence of mitochondrial impairment (Hamano et al., 2014; Tautenhahn et al., 2016). Though single cell RNA sequencing revealed a sophisticated atlas of liver zonation and its spatio-temporal regulation (Halpern et al., 2017; Droin et al., 2021), computational modeling has not yet addressed metabolic zonation in the liver or its dynamic regulation by surgical challenges like partial hepatectomy.

Shear stress as a consequence of hepatectomy-induced portal hypertension seems to be a major trigger of hepatic regeneration and size control, which has been postulated for some time (Sato et al., 1997; Niiya et al., 1999). It has now become clear that LSEC are mainly involved in sensing increased shear stress induced by partial hepatectomy. They respond with an increase in vasoactive compounds like NO (Bartels and Hildebrand, 1975; Poisson et al., 2017), mediated by KLF2-dependent induction of, besides others, NO synthase (Parmar et al., 2006). NO regulates the regenerative response after PHx by orchestrating a cytokine network that includes HGF and IL6 to prime hepatocytes for proliferation and TGFβ as the growth terminating signal (Yagi et al., 2020; de Rudder et al., 2021; Kiseleva et al., 2021). Regulation of liver regeneration after partial hepatectomy by LSEC-mediated mechanical forces was predicted using a computational 3D network of sinusoids substantiating the functional link between hepatic flow control and liver regeneration (Ishikawa et al., 2021).

Flow changes after liver surgery may thus be sensed and transmitted to the cell interior, triggering responses like proliferation or cell migration. Little information is available on whether flow-associated metabolic changes might also be associated with mechano-transduction mechanisms. Yet, there is an obvious and hence likely potential crosstalk between mechano-transduction and molecules involved in metabolic regulation.

#### 2.5.3. β-Catenin Mediates Between Adherens Junctions and Metabolic Imprinting of Perivenous Hepatocytes

β-catenin is part of the adherens junction complex and acts as an intracellular transducer of Wnt (wingless/Int) signaling. It can be mobilized from the adhesion junction complex by tyrosine phosphorylation in response to receptor activation by growth factors like HGF, EGF, and TGFβ. Mobilization can lead to nuclear translocation of β-catenin, activation of genes that promote mitogenesis, and dissociation of the adherens junctions complex required for tissue morphogenesis during regeneration and development (Monga, 2014). Signaling through β-catenin, however, also plays a major role in hepatocyte metabolic specification (Burke and Tosh, 2006; Torre et al., 2011). In tumor cells, high expression of aberrantly active forms of β-catenin coincided with high expression of GS, the key enzyme of ammonia fixation in perivenous hepatocytes (Gebhardt et al., 2007). Providing further evidence, conditional disruption of β-catenin expression in mice caused impairment of ammonia metabolism and perivenous expression of enzymes of the CYP family (Sekine et al., 2006). Activation of Wnt/β-catenin signaling by the GSK3B inhibitor SB-216763 or Wnt-conditioned media increased perivenous marker protein expression in isolated hepatocytes (Hailfinger et al., 2006). Thus, β-catenin is involved in both adhesion junctions and metabolic regulation, predominantly in pericentral hepatocytes.

#### 2.5.4. AMPK Mediates Between Adherens Junctions and Lipid Metabolism

Coordination of tissue homeostasis via adherens junctions and metabolic specification of hepatocytes may be linked via AMPK (Salvi and DeMali, 2018), a central regulator of hepatic lipid metabolism. AMPK is activated through phosphorylation by the upstream kinase complex called LKB1/STRAD-MO25 (liver kinase B1/STE20-related adapter protein-MO25). Activated AMPK in turn inhibits fatty acid synthesis, lipogenesis, and triglyceride synthesis, while anti-lipogenic pathways like fatty acid oxidation and ketogenesis are stimulated (Jansen et al., 2009; Mihaylova and Shaw, 2011). In contrast, dephosphorylation and inactivation of AMPK stimulates fatty acid synthesis, lipogenesis and triglyceride synthesis, which in turn causes steatosis in the chronic stage. Cellular polarity is also regulated by E-cadherin via the LKB1 complex, thus creating a functional link between cell contact maintenance and metabolic regulation of polarized cells such as hepatocytes. In line, the LKB1/STRAD complex and AMPK localize to and thereby stabilize adherens junctions (Sebbagh et al., 2009).

#### 2.5.5. YAP/TAZ Signaling Mediates Tissue Plasticity and Metabolic Regulation

Enhancement of matrix stiffness activates the YAP/TAZ pathway (Dupont et al., 2011). Changes in matrix stiffness are sensed and transduced to the cell interior by the cell adhesion receptors as described above, and communicated to the actin cytoskeleton filaments. Actin reorganization releases cytoplasmic retention of YAP/TAZ. This, in turn, promotes nuclear translocation and transcriptional activation of target genes involved in tissue growth processes like proliferation after injury, embryonic development, and tumor growth in cancer (Pocaterra et al., 2020). Similarly, YAP/TAZ relocation is mediated by substrate stiffness. While soft materials (~1 kPa) foster cytoplasmic localization, stiff substrates (~40 kPa) force nuclear translocation (Dupont et al., 2011; Halder et al., 2012). In the liver, hedgehog-dependent activation of the YAP pathway was necessary to sustain proliferation of hepatocytes after PHx in mice (Swiderska-Syn et al., 2016). The same mechanism also regulated energy supply from glutamine during proliferation of activated HSC. This was associated with epithelial-mesenchymal transition after acute and chronic liver injury (Choi et al., 2009; Du et al., 2018; Chen et al., 2019), indicating a functional relationship between regulation of tissue homeostasis, ECM mechanical properties, and metabolic regulation.

Taken together, mechanical forces induced by changes in hepatic hemodynamics, such as after PHx, appear to play a prominent role in regulating tissue homeostasis and function during liver regeneration, from the organ to the cellular scale.

It should be noted that PHx not only affects the liver but also other organs. In particular, extended liver resections carry a high risk of mortality due to multiorgan failure, indicating an extensive organ-to-organ communication. Cerebral dysfunction is a frequent complication as a result of the increase in neurotoxic metabolites that accumulate due to loss of hepatic detoxification capacity (Søreide and Deshpande, 2021). Although the pathomechanisms of acute kidney injury after hepatectomy remain largely unknown, hemodynamic complications seem to be the prevailing systemic cause of renal complications (Peres et al., 2016). Accordingly, portal hypertension and hyperperfusion in combination with surgery-associated technical procedures like the Pringle's maneuver (occlusion of hepatic artery and portal vein to minimize blood loss) may cause splanchic vasodilation followed by a decrease of the mean arterial pressure, suggesting insufficient perfusion of inner organs including the kidneys (Choukèr et al., 2004; Lee et al., 2009; Tautenhahn et al., 2017). Also, the loss of liver mass after PHx poses *per se* risks to whole body homeostasis due to the decrease in synthetic capacity providing plasma proteins for, e.g., the blood coagulation cascade and the acute phase response, the liver's defense reaction against surgical trauma and inflammation (Ramadori and Christ, 1999), both of which represent potential risk factors after PHx.

### 2.6. Alterations in Gene Expression After Hepatectomy

The state of a cell is mainly determined by its protein composition, which is steadily changing due to protein turnover, i.e., protein degradation and synthesis (Schoenheimer, 1942). An important method to gain information about the cell state is gene expression analysis. However, absolute quantification of gene expression is difficult; therefore, gene expression analysis mostly aims for relative quantification when comparing differences in transcription under various conditions (e.g., age, stress, disease, or surgery).

In the context of liver surgery, gene expression has mainly been analyzed in tissue samples, but more and more single cell studies have recently become available. Although most gene expression studies focus on protein-encoding transcripts, it should be kept in mind that only a small fragment of the human genome is protein coding, and many non-protein coding RNA exist.

Gene expression studies can contribute to the understanding of liver functions in general and liver regeneration after hepatectomy in particular. Recently, gene expression studies have used microarray technology and RNA sequencing to gain new insights into liver regeneration. Multiple studies exist that focus on differential gene expression after surgical interventions like PHx, PVL or ALPPS, with the main model organisms being rat and mouse (see Table 6 for an overview).

**Table 6:** Selection of gene expression studies focussing on hepatectomy and liver regeneration.

**Reference**	**Species**	**Model**	**Study design**	**Results**
**Regeneration after surgery**				
Togo et al. (2004)	Mouse	70% PHx	Microarray	Expression of immediate-early gene candidates (IRAK1, KPNA1), candidate genes during the progress of S1 phase (ID2, ID3), inhibiting factor (GADD45G). NFKB is important in the initial stage of liver regeneration.
Lai et al. (2005)	Rat	70% PHx	Microarray	Expression of many proto-oncogenes changes in the remnant liver during liver regeneration measured 2h to 7 days after PHx. 72 different patterns were identified that describe the change of gene expression.
Cimica et al. (2007)	Rat	70% PHx	SAGE	Strong upregulation of CCND1 16h after PHx. CTGF is induced 4h after PHx. Hepatocyte proliferation occurs 16h after PHx.
Chiba et al. (2013)	Mouse	70% PHx	Microarray	APOA4, HP, FGB and FGG are upregulated during liver regeneration
Li et al. (2018a)	Rat	PVL	Microarray	LLL: activation of hypoxia pathways NLL: activation of cell proliferation and cell-cycle pathways
Rib et al. (2018)	Mouse	2/3 PHx, sham^a^	RNA-Seq	After both sham and PHx, cell-division-cycle genes are activated. 20h after PHx the cell-division-cycle genes are still expressed, but 20h after sham they are not.
**Comparison of age-dependent changes**				
Pibiri et al. (2015)	Mouse	3/4 PHx, sham young, old	Microarray	Similar gene expression in the liver of old and young mice. CCND1 is up-regulated shortly after PHx in young mice, but mostly down-regulated in aged animals. YAP is activated during liver regeneration in elderly, but not in young mice. Other cell cycle genes are expressed similarly in the liver of old and young mice.
Pibiri (2018)	Mouse	PHx young, old		Age-dependent decrease of BubR1, YAP and SIRT1 associated to dampening of tissue reconstitution and inhibition of cell cycle genes after PHx. Reduced liver perfusion may be related to the aging of hepatic stellate cells.
**Comparison of different surgery models**				
Nagano et al. (2004)	Rat	PVL + 90% PHx	Microarray	PVL + 90% PHx vs. sham + 90% PHx: significantly higher survival rate, upregulated gene expression of CCND1.
Borger et al. (2019)	Mouse	ALLPS, 68% PHx, PVL, transection, sham	RNA-Seq	ALPPS vs. 68% PHx: Earlier activation of cell cycle-pathway ALPPS vs. PVL + transection: Enrichment of the IGF1R signaling pathway (cell survival), the ILK pathway (induced cell proliferation), and the IL10 pathway (stability determination), reduced activity of the interferon pathway (transcription).
Colak et al. (2020)	Rat	70% PHx, PVL, ALLPS, sham	RNA-Seq	PHx/ PVL/ ALPPS: Enrichment of cell cycle, mitotic cell cycle, M phase, and DNA replication and repair at 24 h; Downregulation of oxidation-reduction, metabolic process and inflammatory response at 96h. PH/ ALPPS: Downregulation of oxidation-reduction, triglyceride, and steroid metabolic processes PH: Enrichment of cell activation, response to wounding, and immune response
**Mechanosensing**				
Song et al. (2017)				Shear forces and other mechanical cues influence NO signaling, YAP pathway and actomyosin remodeling

*LLL, ligated liver lobes; NLL, non ligated liver lobes; PHx, partial hepatectomy; PVL, portal vein ligation; ALPPS, associated liver partition and portal vein ligation for staged hepatectomy*.

a *placebo surgery (fake operation)*.

#### 2.6.1. Bioinformatical Methods for Differential Transcriptome Analysis

**Figure 3:**
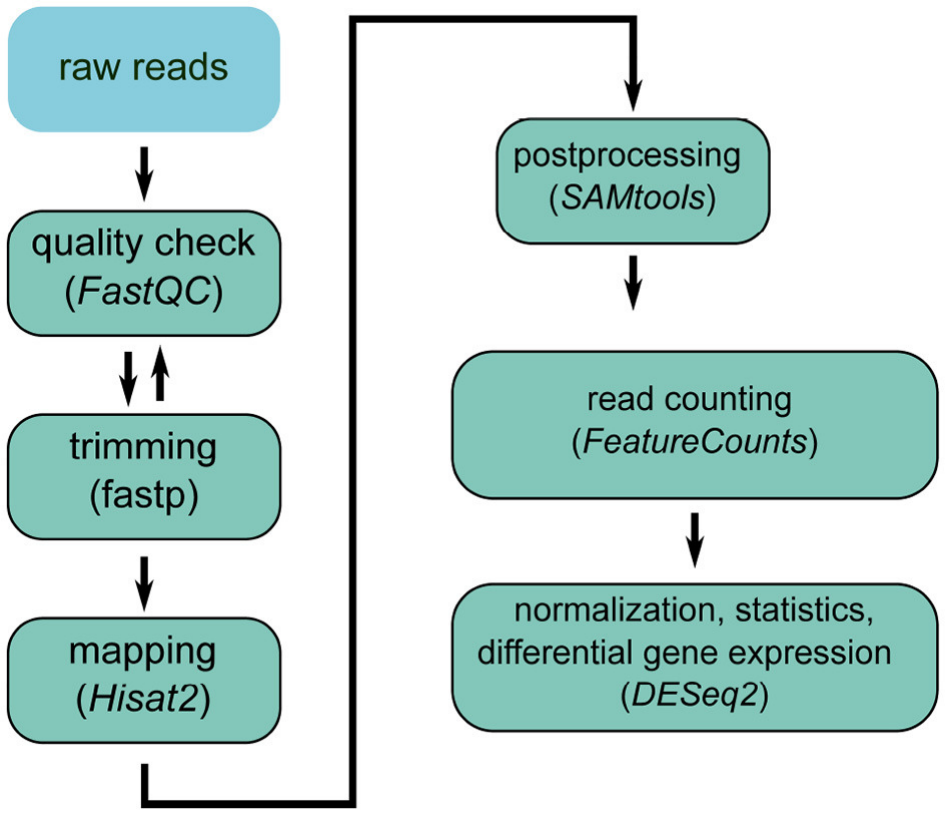
RNA-seq analysis workflow.

In general, the transcriptome is analyzed through RNA-Seq experiments. Figure 3 shows a state-of-the-art workflow. The raw reads, which are produced by sequencing the RNA extracted from the sample of interest, can vary greatly in quality. Thus, the first step during RNA-Seq analysis is quality control (Li et al., 2018a) and trimming, to remove sequencing remainders, e.g., sequencing adaptors. Next, the reads are mapped against the reference genome and post-processed (sorting, conversion to binary format). After counting the reads mapped per gene, the data are normalized to make the results comparable among the different replicates (Love et al., 2014). For differential expression, the normalized gene expression is compared between the different groups (e.g., PHx vs. sham). Thus, a list of differentially expressed genes is returned at the end, which can be used for further downstream analyses, such as pathway analysis or modeling.

Gene expression studies are useful to identify signaling pathways that are most affected by a given procedure. A number of studies have been performed focusing on PHx and confirmed the relevance of cell cycle associated genes in the process of liver regeneration (Borger et al., 2019; Colak et al., 2020). Pathway analyses or gene set enrichment analyses compare differentially expressed genes against pathway databases like KEGG (Kanehisa and Goto, 2000) or GO (Ashburner et al., 2000), in which genes are assigned to different pathways. In this way, the enrichment of complete pathways can be inferred from a list of differentially expressed genes. Examples for liver-specific pathways in the KEGG database are for example hsa05225 [Hepatocellular carcinoma–Homo sapiens (human)], hsa04932 [non-alcoholic fatty liver disease–Homo sapiens (human)] or more general ko04979 (Cholesterol metabolism) and ko01100 (metabolic pathways).

#### 2.6.2. Differential Pathways Activated in Different Surgical Regeneration Models

Comparative studies contribute to the molecular understanding of the different regeneration mechanisms and kinetics observed in different resection models.

Colak et al. (2020) investigated the differential gene expression patterns in rats subjected to PHx, PVL and ALPPS. This study was based on RNA sequencing and provided a comprehensive overview about transcriptomic changes. A similar study by Li et al. (2018a) revealed that hypoxia pathways were activated in the ligated lobes undergoing atrophy. In contrast, cell proliferation and cell-cycle pathways were activated in the non-ligated lobes undergoing regeneration. Nagano et al. (2004) investigated gene expression changes after PVL followed (4 days later) by PHx, compared with rats subjected to PHx alone. The group with PVL had significantly higher survival compared with the control group, which was associated with greater upregulation of CCND1.

Borger et al. (2019) performed transcriptome profiling on two different mouse models to identify pathways associated with liver regeneration. One group underwent ALPPS, while the other group underwent PVL followed by transection. After ALPPS, the IGF1R signaling pathway, the ILK pathway, and the IL10 pathway were significantly enriched, while the interferon pathway was reduced. Furthermore, the PAK- and ILK-associated intracellular signaling pathways were activated at an earlier time point compared to gene expression after 68% PHx only. These findings suggest accelerated liver regeneration after ALPPS. Dupont et al. (2011) already found that YAP expression changes occur in response to changes in tissue rigidity in liver cells. These changes are known to occur with age, but also after hepatectomy. Pibiri et al. (2015) demonstrated strong changes in gene expression of YAP when comparing normal and regenerating liver in young and old mice. Interestingly, YAP expression was significantly upregulated when comparing young and old mouse livers, but not when comparing quiescent and regenerating livers. Pibiri (2018) discussed the possibility of improving hepatic regenerative capacity in the elderly by eliminating senescent cells via autophagy. This hypothesis remains to be confirmed.

#### 2.6.3. Metabolism and Perfusion

Genome-wide expression analysis provides a good basis for identifying differentially expressed pathways. The impact of different surgical methods on gene expression and pathway activation have been well studied (see Table 4). However, most of these studies analyze regeneration after liver surgery in general, without considering the underlying causes separately.

Perfusion, mechanosensing and mechanotransduction are important processes that possibly affect liver regeneration and functional recovery (Song et al., 2017). Only few studies exist that analyze the interplay between these processes (Tchirikov et al., 2002; Nishii et al., 2018).

A comprehensive review of liver metabolism can be found in Rui (2014). More specifically, multiple genes related to metabolism are described to be induced in severe NAFLD. For genes being specifically differentially expressed in the regeneration process after PHx with and without partial portal ligation, we refer to Nobuoka et al. (2006). Notably, minimal CYP reduction was observed during liver regeneration, with the conclusion that portal blood flow plays an important role in liver regeneration and cell cycle gene expression. In another study, shear stress on murine liver progenitor cells could be shown and was reflected by significant upregulation of regeneration-associated genes such as CFOS, IP10, MKP1, ALB, WNT, VEGF, or EpCAM (Nishii et al., 2018).

#### 2.6.4. Integration of Omics Data

In contrast to transcriptomics analysis, which is widely applied in the context of liver surgery, data on changes in proteomics (e.g., Strey et al., 2005; Guo et al., 2006; Sun et al., 2007; Kumar et al., 2013; Chen and Xu, 2014 and metabolomics (e.g., Jung et al., 2013; Samino et al., 2013; Saito et al., 2018; Carril et al., 2020; Zhao et al., 2020) are rather sparse. Almost all studies focus on a single omics approach, while multi-omics has rarely been applied. One exception is the work of Caldez et al. who used an integrated transcriptomic and metabolomic approach to study metabolic remodeling during liver regeneration following hepatectomy (Caldez et al., 2018).

The information from omics studies can be used in combination with computational models to better understand changes in signaling, metabolism and hepatic function. The general workflow for integration is as follows:

(i) Preprocessing and normalization of omics data. In this initial step, data from different experimental conditions and time points are made comparable (normalization of data), outliers are filtered out and a quality control of the data is performed (e.g., removal of batch effects).
(ii) Quantification of differential changes. Most omics approaches provide reliable data for relative changes between conditions, whereas absolute approaches are more complex (e.g., spike ins), often challenging, and rarely applied. The result of the analysis is a list of top molecular candidates based on significance and/or fold change (e.g., a list of differentially expressed transcripts during regeneration after hepatectomy.
(iii) Linking the experimental data with the model. This includes mapping of the experimentally determined fold changes to model parameters and variables in the model. Transcriptomics data is often used as a proxy for changes in protein levels and mapped to the corresponding proteins in the model, whereas proteomics and metabolomics data can be mapped more directly. A computational model for the control condition is often used as a baseline model that is parametrized with the data to obtain multiple model variants corresponding to the various experimental conditions.
(iv) Simulation under differential parametrization. The last step consists of performing simulations with the baseline model and the different model variants and evaluating changes in model predictions.

Since omics data provides information on cellular components (RNA, proteins, metabolites), the corresponding computational models reflect the cellular scale (see section 3.2.1). A classical approach is the parametrization of constraint-based metabolic models to generate tissue-specific or condition-specific models. Examples in the context of liver metabolism are the stratification of patients with HCC based on acetate utilization (Björnson et al., 2015), a metabolic and functional evaluation of NAFLD by integrating metabolic flux data and global transcriptomic data from human liver biopsies (Hyötyläinen et al., 2016), or the study of metabolic pathways in NAFLD (Mardinoglu et al., 2014). Metabolic network-based stratification has been used to reveal distinct tumor subtypes or heterogenous redox responses in HCC using transcriptomics data (Bidkhori et al., 2018; Benfeitas et al., 2019). Benefits of a carbohydrate-restricted diet on hepatic steatosis have been studied using a similar multi-omics approach (Mardinoglu et al., 2018). A similar workflow is applied for kinetic pathway models, for instance to study metabolic heterogeneity in HCC based on proteomics data (Berndt et al., 2021).

The same approach can be applied for spatially resolved data, e.g., using zonated omics. The data is hereby mapped on sub-models corresponding to the respective spatial location (e.g., periportal or perivenous). An example is the study of ammonia detoxification in rodent liver (Bartl et al., 2015).

While omics data have been measured in the context of liver surgery, the integration of these data into computational models is still in its infancy. Time courses of the transcriptome in regeneration exist (different conditions and different time courses), but have not yet been integrated into computational models. Similarly, there are no approaches yet to predict hepatic functions after surgery-induced perfusion changes by computational modeling.

## 3. Data Integration and Computational Modeling

One vision of systems medicine in liver surgery is the development of an integrated modeling framework. This framework should (i) encompass state-of-the-art knowledge of vascular anatomy, tissue architecture, liver perfusion and their impact on key physiological processes, (ii) integrate clinical and experimental data from all relevant measurement/assessment procedures at the different temporal and spatial scales, (iii) allow the study of the system *in silico* using model predictions, and (iv) enable individual risk assessment.

Computational models can generally provide such a framework. In the context of hepatic surgery, the specific goal is to support pre-operative personalized diagnostic and risk assessment. This requires reliable predictions of the residual hepatic function after resection, the hepatic regeneration capacity, and the course of functional recovery after surgery. All three are influenced by perfusion alterations on different scales and their impact on function. In this context, consistent estimation of uncertainties in model predictions is important to inform decisions about surgical strategy (Saltelli et al., 2020).

Achieving this ambitious goal requires a concerted and iterative effort by clinicians, modelers, and experimenters in a modeling-experiment cycle. Such a process typically begins with an initial set of defined experiments and a set of models. In the context of hepatic surgery, these are measurements obtained in cell cultures and animal experiments and in patients undergoing PHx. Multidimensional data include anatomical and morphological data such as hepatic volumetry and information regarding type and severity of pre-existing disease (e.g., steatosis), liver perfusion, quantification of liver function (e.g., by test compounds), and additional data such as omics, all obtained from multiple sources.

Based on existing modeling approaches, structure and granularity of the models must be adapted to the data and the particular question being addressed. For example, assessment of liver function at the organ level does not reflect the heterogeneity of pathways in the liver parenchyma or even in individual hepatocytes, hence appropriate models must be coarse-grained in this regard. Likewise, liver regeneration is observed only on a daily basis, assuming that the time course can be interpolated between measurements on different days. Omics data is gathered from selected ROI, assuming that these **ROI**s are representative of the organ. Integrating data into computational models in such an iterative cycle is generally a challenging task, as is quantifying uncertainty. In this section, we present specific challenges of modeling hepatic perfusion-function relationships for better risk assessment in the context of liver surgery (section 3.1), followed by an overview of relevant computational models on the multiple scales (section 3.2).

### 3.1. Data Integration and Uncertainty Quantification

#### 3.1.1. Lack of Data Integration Standards

Currently, there are no standards for integrating various data types and linking different spatial and temporal scales. In the context of liver surgery, data include (i) imaging data at different spatial scales such as CT and MRI reflecting organ scale and histology reflecting lobular scale, (ii) numerical data on hemodynamic information such as pressure and flow data as well as microcirculation data, (iii) numerical data on metabolism and proliferation reflecting organ and cell scale (see section 2), and (iv) omics data representing molecular scale in a given region of interest. Collectively, these data describe and quantify interrelated processes at the molecular, cellular, lobular and organ scale.

The calibration of models to experimental or clinical data requires a direct comparison between data and model outputs that goes beyond a purely qualitative agreement. Quantification of the quality of a fit in terms of numerical values is necessary for comparison of different model fits and enables the application of optimization algorithms and methods for model comparison. This procedure requires appropriate data pre-processing steps such as normalization, background correction, transformations, elimination of outliers, or estimation of summary statistics. For large datasets, for example omics or imaging data, additional machine learning approaches are used for feature selection and dimension reduction. Often, model calibration is then formulated as an optimization problem in which the pre-processed data are incorporated as numerical values. Such problems belong to the class of (non-linear) inverse problems whose solutions require efficient algorithmic schemes.

For example, a standard modeling approach for hepatic metabolic pathways such as the test compound metabolism or signaling pathways like YAP/TAZ-induced pathways is ordinary differential equations based on chemical reaction kinetics. Time course data of key metabolites are used to estimate unknown reaction rate constants. These data must be normalized and often contain information about fold changes rather than absolute concentrations. This preprocessing step can be done in different ways (Degasperi et al., 2014) and affects sensitivity analysis and summary statistics (Kirch et al., 2016; Thomaseth and Radde, 2016). The objective function in the optimization problem can be the sum of squared differences (least squares estimate) or the likelihood function with an appropriate error model (Kreutz et al., 2007). Coupling metabolic models with models on larger scales, such as models of pressure and blood flow distributions in liver lobules, requires the definition of coupling parameters and, for model calibration, also model reduction techniques.

Standards for representing pathway-based models have been established, with the SBML being the de facto standard (Hucka et al., 2019; Keating et al., 2020). Extensions enable hierarchical model composition (comp package) (Smith et al., 2015), and uncertainty representation (distrib package) (Smith et al., 2020), an important requirement for multi-scale modeling approaches and tracking model and data uncertainty. While mechanisms exist for the annotation of models and data with meta-data (Neal et al., 2019), and how to share experimental and clinical data with the modeling community (König et al., 2021), standards for data-model integration and corresponding workflows are lacking, and coupling models in different mathematical frameworks remains a challenge.

#### 3.1.2. Sparse Data Setting and Uncertainty Quantification

Even if the structure of a model is defined, the data available for model calibration often do not contain sufficient information to unambiguously identify all model parameters. This already applies to models on individual scales, such as intracellular metabolic pathways or pharmacokinetic models at the whole-body scale, due to low time resolution of the measurements or because only a few model components can be quantified. The problem is exacerbated for larger and multi-scale models. Statistical methods generally provide a solution to this problem. They allow consistent tracking of variability in the input data via uncertainty in model parameters to confidence bounds in model predictions. However, many of those methods are computationally expensive and thus not applicable *ad hoc* to larger models, so they must be adjusted accordingly. In general, uncertainty in model predictions is often underestimated because model assumptions are not questioned, the effect of non-modeled factors is neglected, or local sensitivity methods are used even in cases where parameters and input variables are largely uncertain (Saltelli et al., 2020). Since decisions based on model predictions, such as the selection of patients for surgery, are highly dependent on uncertainties, it is important to develop methodology for the adaptation of statistical methods for consistent estimation of sensitivities and uncertainties for the specific problem at hand.

#### 3.1.3. Computational Costs

Coupled processes on different length and time scales, as well as the need for spatial resolution to describe spatial inhomogeneities in the liver, require multi-scale models. These models suffer from long forward simulation times. Hence, standard methods for sensitivity analysis, uncertainty quantification, parameter estimation, or identifiability analysis are not applicable *ad hoc* for these models. For example, a single forward simulation of a spatially-resolved liver lobule on a workstation with i7 processor of the 7th generation, 4 cores and 8 threats requires 15 min to simulate one-twelfth of a single lobule and more than 900 min for a group of seven adjacent lobules. For a global sensitivity analysis, the number of forward simulations to be performed grows exponentially with the number of parameters, resulting in a huge computational cost. Overall, long forward simulation times of multi-scale models combined with difficult inverse problems for model calibration and the need for uncertainty analysis due to sparse data, pose a major challenge toward an integrated framework to support decisions in liver surgery. On the modeling side, there is much room for development of methodology to make the analysis more efficient, such as model reduction techniques or the use of surrogate models to reduce simulation times or efficient numerical schemes to solve optimization problems and quantify uncertainties in model predictions.

#### 3.1.4. Transfer of Models Calibrated With Animal Data to Patients

Another challenge is the translation of models calibrated with animal data to models involving patient data. For example, computational models using hemodynamic measurements after liver resection or **PVL**s in rodents need to be adapted to the patient situation, taking into account different anatomical features as well as parameters like age, gender, and pre-existing diseases. It is known that animal studies are often poor direct predictors of human responses to medical treatments or exposures (Perel et al., 2007; Bracken, 2009). A modeling framework adapted to human parameters could address this problem, but is challenging. Not only does one have to deal with different types of data for humans and animals, human patients have much larger variability. This is because liver (dys)function, liver metabolic status, and function-perfusion relationships depend on individual lifestyle, environmental influences, and dietary habits. The effects of those factors can be controlled in designed animal studies. Thus, building predictive models for patients undergoing liver surgery based on models fitted to animal data is a multifaceted problem. However, it is also known that many physiological and mechanical parameters, such as lobulus architecture or liver perfusion, are similar in humans and can therefore be transferred (Kruepunga et al., 2019). For some parameters, such as the regeneration course after resection, transfer can be done by proper rescaling of the time scale, for which comparison studies are available in the literature (Periwal et al., 2014). If a transfer of parameters is not possible, models must be adapted to patients by calibrating selected parameters to human data. Furthermore, models can be enriched by analysis methods applied to human data to extract influential features on regeneration trajectories as well as risk analyses and their integration into models.

### 3.2. Computational Modeling

**Figure 4:**
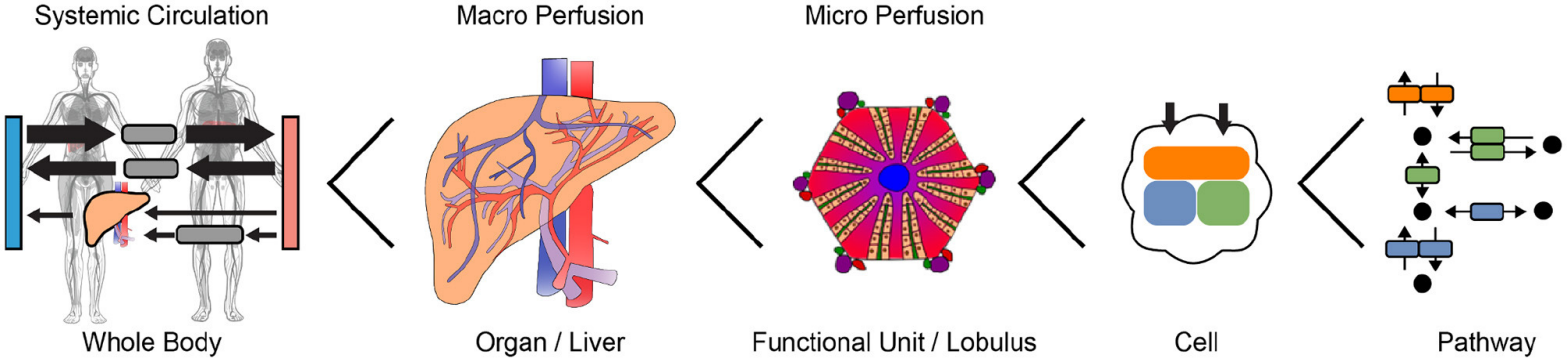
Multiple scales relevant for computational modeling of perfusion-function relationships. Anatomograms under CC-BY Papatheodorou et al. (2020).

Multiscale computational models are a unique approach to gain a better understanding of liver surgery-induced alterations in hepatic perfusion, function and subsequent regeneration. The relevant scales range from whole body to single cell (Figure 4). At the whole-body scale, the systemic circulation connects the liver to the rest of the body. At the organ scale, blood is distributed within the liver through a network of hepatic vessels (macroscopic perfusion) that supply the liver with oxygen-rich blood from the hepatic artery and nutrient-rich blood from the portal vein. The hexagonal liver lobules form the functional units of the liver, in which the blood is guided via sinusoids along the liver cells from the outer periportal region to the central perivenous region (microcirculation). The perivenous blood is subsequently drained from the liver into the inferior vena cava via the hepatic veins. Mechanotransduction, signal transduction, and metabolism occur at the level of the hepatocytes located along the blood vessels (sinusoids) of the liver lobules.

In this section, we review relevant computational models for understanding the effects of hepatectomy-induced alterations in perfusion on hepatic function at the cellular scale (section 3.2.1), lobular scale (section 3.2.2), organ and whole-body scale (section 3.2.3).

#### 3.2.1. Cellular Scale

**Figure 5:**
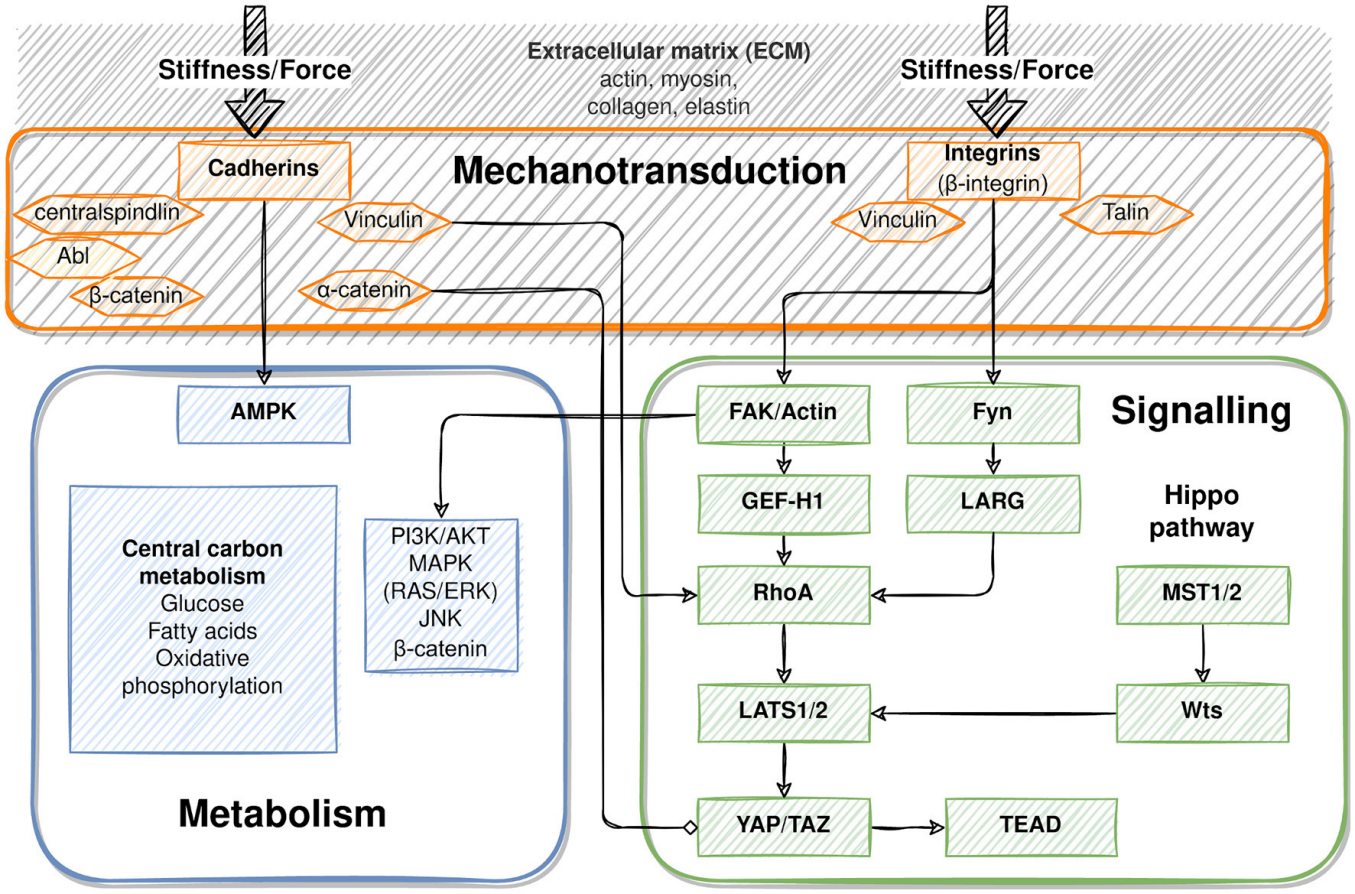
Cellular adaptations to changes in tissue perfusion and mechanical properties (mechanotransduction, signaling, metabolism). The extracellular matrix (collagen, proteoglycans) plays an important role in how changes in perfusion and pressure are translated to changes in stiffness and force. Changes in stiffness and force are sensed by cadherins and integrins (mechanotransduction), which transfer the information via signaling cascades. A main signaling route is the FAK to YAP/TAZ pathway, with important crosstalk from the Hippo pathway. Important signaling pathways regulating metabolism can be modified due to mechanotransduction, among them AMPK, PI3K/AKT, MAPK, JNK and β-catenin which regulate central metabolic functions. See section 2.5 for details.

Computational models at the cellular scale can provide insights into the mechanism of how changes in tissue micro-perfusion and mechano-properties can alter hepatic function. Relevant processes include mechanotransduction, signaling, and metabolism (Figure 5); selected computational models are listed in Table 7.

**Table 7:** Selection of computational models on cellular scale.

**Reference**	**Mechano transduction**	**Signaling**	**Metabolism**	**System**	**Approach**	**Geometry**	**Result**
Nikmaneshi et al. (2020)	✓		✓	Mechanotransduction to metabolism (empirical shear stress response).	ODE, FEM	Sinusoid/ lobulus	Mechanobiological mathematical model of liver metabolism. Modeling empirically the effect of shear stress on urea and albumin synthesis through mechanotransduction mechanisms.
Peng et al. (2017)	✓	✓		Stiffness sensing, mechanotransduction; signaling (YAP/TAZ);	ODE	Mesenchymal stem cell	Mathematical model of mechanotransduction. Modeling how mechanical memory regulates mesenchymal stem cell fate decisions.
Guberman et al. (2020)	✓	✓		ECM, mechanosensing, YAP, PI3K, AKT, MAPK.	Boolean	Epithelial cell	Boolean regulatory network model that synthesizes mechanosensitive signaling that links anchorage and matrix stiffness to proliferation and migration.
Gérard and Goldbeter (2014)	✓	✓		Mammalian cell cycle integrated with ECM via FAK and contact inhibition via Hippo/YAP pathway.	ODE	Mammalian cell	Model of cyclin-dependent kinases (CDK, cell cycle) with phenomenological effect of ECM via FAK and cell density (contact inhibition mediated via Hippo/YAP pathway).
Meyer et al. (2020)	✓	✓		Modeling (zonated) nuclear YAP levels following mechanical stimulation.	ODE/ PDE	Cell/lobule	Mechanistic model for switch-like activation of YAP upon mechanical stimulation during regeneration in the context of bile acid overload.
Sun et al. (2016)	✓	✓		YAP/TAZ signaling and mechanosensing.	ODE	Cell	Model of YAP/TAZ mechanosensing converting extracellular-matrix mechanical properties to biochemical signals via adhesion, and integrating intracellular signaling cascades associated with cytoskeleton dynamics.
Labibi et al. (2020)		✓		YAP/TAZ signaling	ODE	Cell	Model of TGF-β/Smad nuclear accumulation by YAP/TAZ.
Shin and Nguyen (2016)		✓		Hippo signaling (MST1/2, LATS 1/2)	ODE	Cell	Model of Hippo-ERK signaling.
Scott et al. (2020) (preprint)		✓		YAP/TAZ translocation via stiffness transfer function (RhoA, Fak)	PDE	cell	Spatial modeling of YAP/TAZ nuclear translocation.
Wehling et al. (2021) (abstract)		✓		YAP/TAZ shuttling	PDE	Cell	Mathematical model of YAP and TAZ nuclear/cytoplasmic shuttling in liver cancer cells using 2D reaction diffusion equations.
Aprupe et al. (2020) (abstract)		✓		YAP/TAZ shuttling and Hippo pathway activation	ODE	Cell	Mathematical model of the YAP/TAZ shuttling as a response to cell density, actin dynamics, and liver damaging drugs.
Gille et al. (2010)			✓	HepatoNet1 - Genome scale metabolism.	CB	Hepatocyte	A comprehensive metabolic reconstruction of the human hepatocyte for the analysis of liver physiology.
Mardinoglu et al. (2014)			✓	iHepatocytes2322 - Genome scale metabolism.	CB	Hepatocyte	Genome scale metabolic model of hepatocyte.
Berndt et al. (2018)			✓	Central carbon metabolism	ODE	Hepatocyte	A biochemistry-based model of liver metabolism.
König and Holzhütter (2012); König et al. (2012)			✓	Glucose metabolism	ODE	Hepatocyte/ liver	A detailed kinetic model of human hepatic glucose metabolism and application to disease.

*ODE, ordinary differential equations; PDE, partial differential equations; ABM, agent-based model; CB, constraint-based approach (flux balance analysis); DCM, deformable cell models*.

Mechanotransduction is the process of sensing and translating changes in stiffness/elasticity and force into a cellular signal (see also section 2.5). Substantial knowledge exists on the biochemical and mechanical properties of the ECM (collagen, proteoglycans) and its interaction with the cytoskeleton (actin, intermediate filaments). Detailed computational models have been developed on these interactions (see Elosegui-Artola et al., 2018 for a recent review). Less information and models exist on mechanotransduction via integrins and cadherins. Examples range from a model that couples mechanotransduction to YAP/TAZ signaling (Peng et al., 2017), to a model of how ECM mechanical properties convert to biochemical signals via adhesion and integrated intracellular signaling cascades associated with cytoskeleton dynamics (Sun et al., 2016), to a mathematical model of Hippo and TGFβ cross-talk to YAP/TAZ signaling (Labibi et al., 2020).

Signaling pathways translate the mechanical signals into cellular responses. Existing models representing mechanosensing and mechanotransduction mainly focus on the YAP/TAZ pathway, e.g., YAP/TAZ mechanosensing (Sun et al., 2016) or the control of TGFβ/Smad nuclear accumulation by YAP/TAZ (Labibi et al., 2020). An important part of the YAP/TAZ signaling is the shuttling of YAP/TAZ between nucleus and cytoplasm. Scott et al. modeled this nuclear translocation via a stiffness transfer function (Scott et al., 2020), and Wehling et al. used 2D reaction diffusion equations (Wehling et al., 2021). In contrast, Aprupe et al. applied an ODE approach to model YAP/TAZshuttling as a response to cell density, actin dynamics, and liver damaging drugs (Aprupe et al., 2020). Meyer et al. presented a mechanistic model predicting switch-like activation of YAP upon mechanical stimulation during regeneration (Meyer et al., 2020). Guberman et al. (2020) developed a Boolean regulatory network model in epithelial cells that synthesizes mechanosensitive signaling linking anchorage and matrix stiffness to proliferation and migration, including key signaling players such as YAP, PI3K, AKT, and MAPK.

Hence, YAP/TAZ has been the focus of numerous modeling approaches, but linkage and crosstalk to other signaling cascades is still in its infancy. One such example is a model of CDK with phenomenological effect of ECM via FAK and cell density (contact inhibition mediated via Hippo/YAP pathway) (Gérard and Goldbeter, 2014). Metabolism encompasses the set of chemical reactions, with the liver being the metabolic hub of the body. The activity of a key regulator of cell metabolism, AMPK, has been shown to be mechanoresponsive and thus can bridge adhesion mechanotransduction and energy homeostasis (Isogai et al., 2017).

Consequently, key hepatic pathways such as glucose metabolism, fatty acid metabolism, or oxidative phosphorylation can be affected by changes in perfusion. Similarly, many of the pathways relevant for evaluating liver function using test compounds, such as cytochrome P450 detoxification (e.g., LiMAx via CYP1A2) or transport and secretory activity (e.g., ICG) could be mechanosensitive and be affected by perfusion changes following hepatectomy. Metabolic models of many of these pathways have been established in recent years. Genome-scale models of hepatic metabolism using a constraint-based approach exist (Gille et al., 2010; Mardinoglu et al., 2014) as do detailed kinetic models of central carbon metabolism (Berndt et al., 2018) or glycolysis (König and Holzhütter, 2012; König et al., 2012). Small metabolic pathway models are often included in lobular and organ scale models, e.g., acetaminophen detoxification (Sluka et al., 2016; Fu et al., 2018; Means and Ho, 2019) or glucose metabolism (Ricken et al., 2015). However, linking metabolic pathway models to mechanotransduction and mechano-signaling has rarely been established. One such example is a mechanobiological model to study the effect of shear stress on urea and albumin synthesis (Nikmaneshi et al., 2020).

In summary, models have been established for key signaling pathways involved in mechanotransduction as well as metabolic models for pathways important for liver function, such as central carbon metabolism or detoxification. However, models integrating mechanosensing/mechanotransduction with these signaling pathways and subsequent changes in hepatic metabolism are lacking so far. Furthermore, most of the existing models on mechanotransduction and signaling are not liver-specific.

#### 3.2.2. Lobular Scale

In recent years, a wide range of computational models for simulating hepatic perfusion at the liver lobule scale has been presented (Table 8). These models differ both in the selection of the method and in the consideration of perfusion processes. An overview of computational models for hepatic processes can be found in Christ et al. (2017) or Ricken and Lambers (2019). A common method for describing hepatic microcirculation at the lobular scale is CFD. Using this method, simulations of hemodynamics in a portion of a lobule (Rani et al., 2006) or one respective sinusoid (Ma et al., 2020) have been presented. Extension of the geometry to a hexagonal shaped liver lobule (Debbaut et al., 2014) using CFD has provided insights into the spatial distribution of lobular perfusion and microcirculation.

**Table 8:** Selection of computational models on lobular scale.

**Reference**	**Perfusion/ Permeability**	**Function**	**Modeling approach**	**Geometry**	**Result**
Debbaut et al. (2014)	Vascular perfusion (Darcy flow), anisotropic permeability	No function	CFD, porous medium approach	Hexagonal liver lobule	This simulation examines the importance of vascular septa for blood perfusion in liver lobules. It takes into account hepatic isotropic and anisotropic permeability.
Rani et al. (2006)	Vascular perfusion (3D- Navier Stokes), shear-thinning model	No function	CFD	Blood vessel, no lobule	Simulation of blood flow in hepatic lobule, distinction between arterial and portal venous blood, pressure values and velocity at different vessels
Bonfiglio et al. (2010)	Newtonian fluid plus shear-thinning model, Darcy flow	No function	Porous medium approach	Hexagonal liver lobule	The model simulates changes in blood perfusion after resection using anisotropy and shear-thinning modification.
Antonov et al. (2017)	Weekly compressible Newtonian fluid, Darcy flow with continuity assumption	No function	Double porosity model, porous medium approach	Hexagonal liver lobule	A model to quantify the pressure distribution in a liver lobule
Hu et al. (2017a)	Darcy flow for incompressible Newtonian fluid	No function	Porous medium approach	Hexagonal liver lobule	A mathematical model for flow dynamics in normal, fibrotic and cirrhotic livers. The model illustrates pressure distribution that is validated with experimental data from rats.
Ma et al. (2020)	1D Navier Stokes equation	No function	1D Navier Stokes equation for blood flow	Vascular tree pre- and postoperative	The simulation of preoperative and postoperative perfusion and postoperative hepatic hemodynamics after left hepatectomy
Sluka et al. (2016)	Multicell modeling package CompuCell3D (CC3D)	Acetaminophen metabolism	Multiscale model: (PBPK modeling at whole body scale, Multicell (CC3D) at tissue/ organ scale, reaction kinetics at sub-cellular scale)	Single sinusoid, single hepatocyte	The multiscale model combines all scales as standalone models. Along one sinusoid the APAP plasma concentration is simulated at all scales.
Fu et al. (2018)	Hagen-Poiseuille's law with mass conservation with advective-diffusive transport processes	Xenobiotic metabolism	Transport and metabolism approach in sinusoidal network	Hexagonal shaped lobule as network of compartments based on experiments	This model investigates the spatio-temporal xenobiotic and metabolite concentration with zonation along one hexagonal shaped liver lobule.
Berndt et al. (2018)	Hagen-Poiseuille law for fluid flow through a cylinder	Glucose metabolism	Compartment model with metabolic model	Sinusoidal tissue unit (STU)	Simulation of zonated carbohydrate metabolism, hepatic glucose exchange and hormone clearance. Investigation of metabolic differences due to inhomogeneous blood perfusion in a sinusoid.
Hoehme et al. (2020)	No perfusion	Liver regeneration after PHx / detoxification	Hepatocytes: isotropic, elastic, adhesive object cabable of active migration, growth and division cell interaction: JKR-force model	Liver lobe examined from mouse/pigs experiments with hepatocytes as individual modeling units	A predictive computational model shows that biomechanical cell cycle progression control can explain liver regeneration after partial hepatectomy.
Boissier et al. (2020)	Poiseuille law	Detoxification	Modeling microcirculation/ blood flow and convection-reaction	Hexagonal liver lobule	A model for the simulation of hemodynamics and advection-reaction transport processes to model the detoxifiying organ function.
Diaz Ochoa et al. (2012)	Liquid with a low Reynolds number, Einstein relation	Acetaminophen metabolism	Multiscale model	Hexagonal liver lobule	Simulation of spatial distribution of APAP concentration and cell viability
Ricken et al. (2010, 2015); Ricken and Lambers (2019)	Darcy flow	Glucose and fat metabolism	Theory of Porous Media, Mulitscale model (PDE on lobule scale, ODE on cell scale)	Hexagonal liver lobule	Simulation of spatial distribution of glucose and lipid concentration, spatial distribution of blood perfusion, interplay between perfusion and function, accumulation of fat with influence on blood flow and growth processes

*CFD, computational fluid dynamics; 3D, three-dimensional; 1D, one-dimensional; PBPK, physiological-based pharmacokinetics model; PDE, partial differential equations; ODE, ordinary differential equations*.

The porous structure of the liver and the resulting effects are captured by porous media approaches. Various models have been developed to simulate lobular perfusion (Antonov et al., 2017) after liver resection (Bonfiglio et al., 2010) or in fibrotic and cirrhotic liver (Hu et al., 2017a).

However, these models do not consider hepatic function and its coupling with perfusion. Several multiscale models combine processes on organ, lobular and cellular scale using PBPK equations (Diaz Ochoa et al., 2012; Sluka et al., 2016) or PDE-ODE coupling (Ricken et al., 2015).

To mimic the behavior of hepatic processes at the lobular scale, a detailed knowledge about the organ physiology as well as the sensitivity to changes in boundary conditions is required. A suitable simulation of function-perfusion-coupling depends on material parameters as well as boundary conditions used as initial values for the lobular model. Since the model quality is directly related to the quality of the available data, detailed observation of hepatic conditions and their measurements are necessary. Information on liver geometry, heterogeneity of liver lobules, damage such as the degree of fatty tissue, hyperperfusion, growth and remodeling (Ricken et al., 2007; Ateshian and Ricken, 2010; Ricken and Bluhm, 2010) and the degree of resection must be determined in experiments or clinical procedures and then integrated into the computational framework.

For example, computational modeling at the lobular scale requires information about tissue elasticity to capture the poroelastic behavior. Information on the mechanical behavior of liver tissue can be determined using, e.g., indentation methods or multiparametric MR elastography (Seyedpour et al., 2021). The elasticity modulus can be determined non-invasively via various methods described in Section 2.3.1. To capture the realistic geometry of liver lobules with the respective inflow and outflow conditions, the geometry can be determined by image segmentation from histological sections (Ahmadi-Badejani et al., 2020). Hepatic blood flow can be measured using Doppler-US or functional MRI for macroscopic blood flow as well as OPS for microcirculation (Ricken et al., 2010).

The development of a robust and efficient multiscale model must integrate the data provided by cellular models via appropriate coupling parameters. To reduce computational costs, MOR techniques (Armiti-Juber and Ricken, 2021) can be implemented. To get an initial intuition of the model properties, surrogate models of parts of the simulation are compiled for a quick analysis of the component behavior.

#### 3.2.3. Organ and Whole-Body Scale

**Figure 6:**
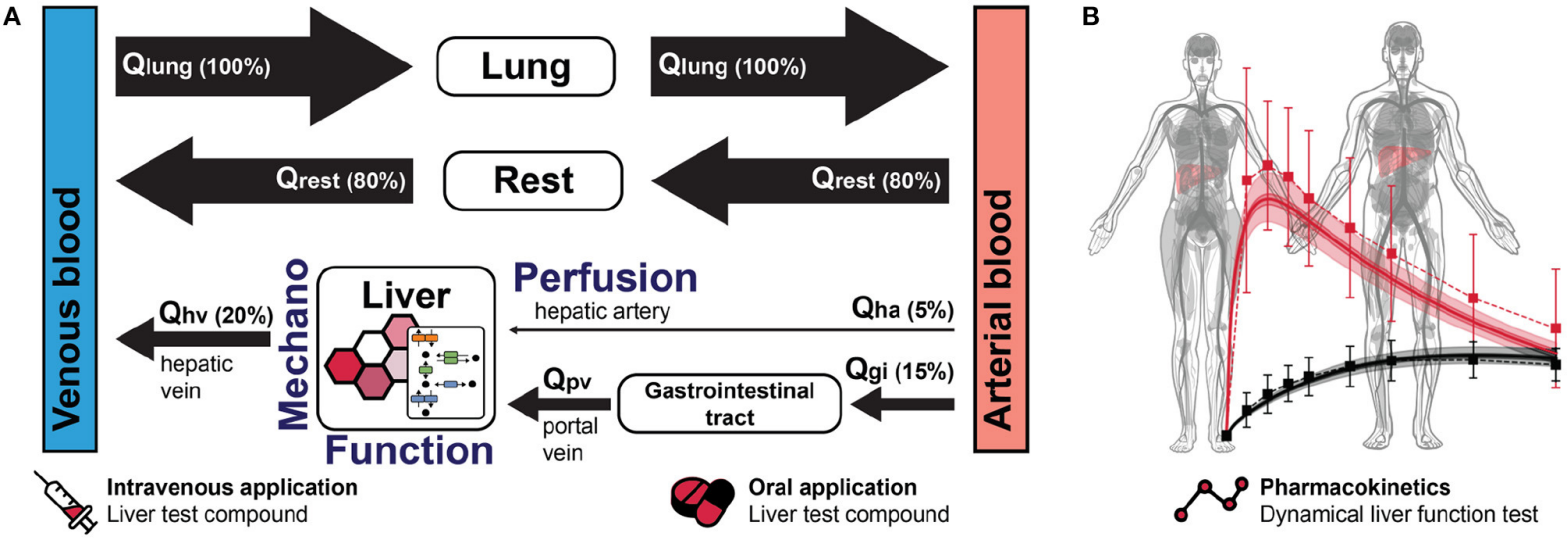
Physiologically based pharmacokinetic (PBPK) model for liver function at the whole-body scale. **(A)** PBPK models include organs and the systemic circulation between these organs. The liver receives approximately 20% of the cardiac output as blood flow with one-quarter from the hepatic artery and the remainder from the portal vein. Liver function depends on hepatic perfusion and metabolism, as well as on mechanical tissue properties, which can influence hepatic function either directly *via* changes in blood flow or indirectly by changes in metabolism following mechanotransduction. In the example, all organs besides the liver and the lung have been pooled in the Rest compartment for simplicity. Q_lung_, blood flow lung; Q_ha_, blood flow hepatic artery; Q_gi_, blood flow gastrointestinal tract; Q_pv_, blood flow portal vein; Q_hv_, blood flow hepatic vein; Q_rest_, blood flow rest of body. **(B)** The pharmacokinetics of liver test compounds, as measured in dynamic liver function tests such as ICG elimination or LiMAx, allows assessment of liver function at the whole body scale. Shown here in red caffeine (test compound) and in black paraxanthine (main product) in an example liver function test based on caffeine. PBPK models are excellent for modeling these tests. Anatomograms under CC-BY Papatheodorou et al. (2020).

Computational models at the whole-body scale can provide important information on systemic circulation and hepatic blood flow, as well as whole-body function. PK models can provide an accurate description of whole liver clearance or metabolization/function (Willmann et al., 2003). An important class are PBPK models describing the whole body (Jones and Rowland-Yeo, 2013) (Figure 6). These models include the systemic circulation between organs and are therefore uniquely suited to evaluate the effect of blood flow changes on hepatic function. PBPK models integrate information from multiple sources, including drug-dependent, physiological, and biological parameters and their variation between species, subjects, or with age and disease state. The biological and mechanistic bases of PBPK models allow extrapolation of kinetic behavior between species (Espié et al., 2009).

Various hepatic clearance models have been developed to integrate the factors of blood flow, binding, transport and metabolism at the whole-body scale to predict clearance of substances (Ho and Zhang, 2020). The simplest model is the well-mixed model, which describes the liver as a single compartment in which the drug concentration in the liver is in equilibrium with that in the emergent blood and the concentration of compounds is identical throughout the liver (Rowland et al., 1973; Pang and Rowland, 1977). Approaches to describe heterogeneity within the liver include the parallel tube model (Winkler et al., 1973), the distributed-model (Bass et al., 1978), the series-compartment model (Gray and Tam, 1987), and the variable transit-time or dispersion model with mixing between sinusoidal blood and hepatocytes (Goresky et al., 1973).

While PBPK models are routinely applied to predict drug detoxification under healthy and disease conditions (e.g., liver cirrhosis Edginton and Willmann, 2008), their application in the context of liver surgery and hepatectomy is still in its infancy (Table 9). Existing models account for the loss of liver volume in hepatectomy by reducing the liver volume *in silico* (Lagneau et al., 2005; Lu et al., 2006; Köller et al., 2021a,b) and allow simulations of the effects of different resection rates on pharmacokinetics (e.g., on liver function measured by ICG Köller et al., 2021a,b). The model by Lagneau et al. considered changes in perfusion or hemodynamics due to liver surgery, i.e., intraoperative liver pedicular triad clamping, intraoperative blood loss, and the associated changes in cardiac output in addition to the volume loss (Lagneau et al., 2005).

**Table 9:** Selection of computational models on organ and body scale-related to hepatectomy.

**Reference**	**Approach**	**Species**	**Geometry**	**Result**
**Function (pharmacokinetics)**				
Köller et al. (2021b)	PBPK	Human	Whole-body	PBPK model of ICG pharmacokinetics in hepatectomy. Risk assessment based on pre-operative ICG parameters.
Lu et al. (2006)	PBPK	Rat	Whole-body	PBPK model of hexachlorobenzene pharmacokinetics after partial hepatectomy.
Lagneau et al. (2005)	PBPK	Human	Whole-body	PBPK model of cefazolin pharmacokinetics in (right) hepatectomy) accounting for (i) intraoperative liver pedicular triad clamping; (ii) intraoperative blood loss; (iii) reduction of liver volume after resection.
Thomas et al. (2015)	Linear regression	Human	Whole-body	Linear regression model. Post-resection liver volume could be accurately predicted by the time course of liver inflow, but not by resected liver volume.
**Haemodynamics**				
Ho et al. (2010, 2012)	CFD	Human	Liver	Hemodynamic model for a patient who received a right donor lobectomy. Hemodynamic equations were solved subject to the sonographically measured inlet velocity and models of portal veins via MRI/CT.
Ma et al. (2020)	CFD	Human	Liver	Hemodynamic model after left hepatectomy (1D Navier-Stokes). Exploration of post-hepatectomy outflow boundary conditions.
Lin et al. (2021)	CFD	Human	Liver	Highly parallel method to simulate blood flows in the liver including hepatic artery, portal vein and hepatic vein from CT data (transient incompressible Navier-Stokes equations). Simulation of pressure, velocity and WSS in hepatectomy.
Debbaut et al. (2012)	Closed loop 0D	Rat	Whole-body	An electrical rat liver model to compare normal with resected liver hemodynamics. Lobe-specific resistive lumped parameter model of the liver. Results demonstrated hyperperfusion effects such as portal hypertension and elevated lobe-specific portal venous flows.
Audebert et al. (2017)	Closed loop 0D	Pig	Whole-body	Closed-loop lumped parameter model for hemodynamics changes observed during hepatectomy in pig. Increase in portal pressure, increase of liver pressure loss, slight decrease of portal flow and major decrease in arterial flow are quantitatively captured.
Golse et al. (2021)	Closed loop 0D	Human	Whole-body	Closed loop 0D model to anticipate postoperative hemodynamics with good correlation between measured and simulated portal vein pressure.

*PBPK, physiological-based pharmacokinetics model; CFD, computational fluid dynamics; 0D, zero-dimensional; 1D,one-dimensional*.

Hemodynamics is included in PBPK models in the form of blood flows between compartments and tissues, but important information such as pressure, pressure gradients, or tissue resistance is missing. In contrast, approaches simulating CFD allow detailed capture of postoperative hepatic hemodynamics in reconstructed vascular geometries. CFD models have been applied to study the changes in hemodynamics following hepatectomy in humans (Ho et al., 2010, 2012; Ma et al., 2020; Lin et al., 2021). CFD approaches can be computationally very intensive (Lin et al., 2021). Moreover, the existing examples only cover the respective reconstructed vascular structures of the liver, without information about the systemic circulation. Closed loop 0D models (a lumped parameter model of the entire circulation without any spatial dimensions) allow detailed modeling of cardiac output and systemic circulation (Shi et al., 2011). Depending on their resolution, the liver can either be modeled as a single unit or the structure of the lobes can be resolved. Closed loop 0D models can be solved efficiently, reflect physiology, and be used to model surgical interventions. Such models have been applied to study the impact of PHx on the hepatic hemodynamics in rats (Debbaut et al., 2012), pigs (Audebert et al., 2017), and humans (Golse et al., 2021). 0D models can easily be coupled with 1D, 2D, or 3D models of vessels or tissues and contain information on pressures and flows, both important requirements for multi-scale approaches. Systemic circulation has been studied in detail with 0D models, but coupling with PBPK models to describe clearance of substances through the liver has not been done to our knowledge.

An important step would be to couple models of hemodynamics (0D closed loop) and function (PBPK) at the whole-body scale with spatially resolved 1D/2D/3D approaches on the sinusoid/lobule scale. Application of such multi-scale models to dynamic liver function tests (such asICG or LiMAx) could provide important insights in how alterations in hepatic hemodynamics after hepatectomy affect liver function.

The liver can only perform its various functions in the context of the entire body, and conditions affecting other organs can alter liver function. Heart disease is often associated with altered cardiac output, resulting in changes in hepatic perfusion, which affects the clearance of substances by the liver. The marked effects of changes in cardiac output on liver function as measured by ICG were studied with a recent PBPK model in Köller et al. (2021a). Simulations of blood flow using computational models of the heart could provide important insights into the heart-liver connection. Kidney disease resulting in renal impairment can have effects on liver function due to changes in clearance of substances by the kidneys, leading to an additional burden on the liver. Furthermore, renal impairment is often associated with alterations in blood pressure, resulting in changes in systemic circulation. Comorbidities of other organs can also affect the results of liver function tests, e.g., pulmonary disease can alter the results of respiration tests such as the LiMAx test. While some hepatic functions are affected by a few organs (e.g., ICG by the heart and changes in cardiac output), other functions such as carbohydrate and energy metabolism, which play a crucial role in regeneration after hepatectomy, can be affected by multiple organs (e.g., insulin secretion by the pancreas, glucose utilization by muscle, free fatty acid release by adipose tissue, glucose filtration by the kidneys, …).

PBPK models provide a unique platform to understand how organs besides the liver and comorbidities of these organs can affect hepatectomy and regeneration after hepatectomy. Important future work, not only in the context of liver surgery but for the systems medicine approach in general, will be the integration of computational models of various organs toward an *in silico* human.

## 4. Perspective

In this review, we have delineated current knowledge on the relationships between alterations in hepatic perfusion and their consequences for hepatic function in the context of liver surgery, using hepatectomy as an example.

Computational models and systems medicine approaches can contribute to a better understanding of the complex perfusion-function interactions after hepatectomy at all relevant spatial and temporal scales, from the single cell to the whole liver and from immediate changes after surgery to long-term regeneration. Multiscale models provide a unique opportunity to integrate heterogeneous experimental and clinical data. But despite a wide array of available tools in clinical and experimental settings, data generated with these methods have not yet been systematically integrated into multi-scale computational models in the context of liver surgery. Major challenges remain, such as lack of data integration standards, sparse data settings and uncertainty quantification (Pivovarov et al., 2019), computational cost of multi-scale models, and transfer of models calibrated with animal data to patients. Key requirements for success are tight cooperations between animal, clinical, and modeling research in an iterative cycle. Especially important in this context is the generation of animal data orthogonal to the patient data for model calibration, complemented with data featuring sufficient overlap between animals and patients to enable transfer.

Current computational tools applied in the clinic are limited to visualization of tumor localization in relation to individual vascular anatomy and virtual planning of the resection plane. Virtual resection allows visualization and quantification of the volume of the territories at risk. However, current visualization tools cannot determine the impact of impaired hepatic venous drainage on hepatic function. They do not account for the impact of surgically induced perfusion perturbations on hepatic function after resection. A key challenge is to develop multi-scale computational models for liver function diagnostics applied in liver surgery, such as the dynamic liver function tests based on ICG or LiMAx. Currently, there is no integrated cross-scale computational model that integrates functional aspects at the molecular and cellular level as well as hepatic perfusion at the lobular level to predict global function at the organ level. Therefore, predictions that account for any spatial inhomogeneity are not possible. Multidimensional modeling is needed to bridge the gap between current possibilities to assess hepatic perfusion, metabolism and regeneration and the need for reliable predictions for individual patients.

A systems medicine approach based on multiscale predictive models and incorporated into the clinical decision-making process could provide actionable information. In the context of liver surgery, the use of these techniques will improve prediction of outcomes after extended liver resection. This may help avoid surgery in high-risk patients and enable a personalized approach to advanced hepatic surgery. Based on the models and resulting predictions, individual pre-rehabilitation interventions can be initiated to reduce perioperative morbidity and mortality after extensive liver resections.

To be successful, two major gaps need to be addressed: (i) the application of multi-scale models in the context of liver surgery and (ii) the consideration of the complex perfusion-mechano-function interactions at the micro- and macro-scale using such a systems medicine approach.

Future clinical translation is facing even higher complexity. Extrinsic factors and processes involving individualized patient data comprising underlying liver diseases and systemic comorbidities must be included. The high complexity of the data needs advanced modeling and data integration strategies that require concerted iterative actions between experimenters, clinicians, and modelers. In this sense, this review aims to promote mutual understanding of biological processes and their computation in systems approaches by natural scientists, physicians and modelers to pave the way for future systems medicine implementation in clinical practice. This also envisions collaborative efforts to couple systems medicine models with existing hospital information systems to enable interoperability and integration of patient data and predictive models. Our long-term vision is an application integrated in the hospital information system that enables accurate estimation of liver regeneration and postoperative morbidity and mortality already at the virtual planning stage of surgery, with the goal of establishing reliable individualized and patient-tailored liver surgery.

## Author Contributions

BC, UD, K-HH, MK, LL, MM, NR, JRR, TR, and H-MT were involved in design ing, writing and revising the manuscript. MC, SH, and DM wrote sections of the manuscript. All the authors contributed to manuscript revision, read, and approved the submitted version.

## Funding

This work was supported by the German Research Foundation (DFG) within the Research Unit Programme FOR 5151 QuaLiPerF (Quantifying Liver Perfusion–Function Relationship in Complex Resection—A Systems Medicine Approach) by grant no. 436883643. NR, TR, and LL are supported by Deutsche Forschungsgemeinschaft (DFG, German Research Foundation) under Germany's Excellence Strategy–EXC 2075–390740016. UD, TR, and H-MT are supported by Deutsche Forschungsgemeinschaft (DFG, German Research Foundation) by grant no. 465194077 (Priority Programme SPP 2311, Subproject SimLivA). TR is supported by Deutsche Forschungsgemeinschaft (DFG, German Research Foundation) by grant no. 312860381 (Priority Programme SPP 1886, Subproject 12). MK is supported by the Federal Ministry of Education and Research (BMBF, Germany) within the research network Systems Medicine of the Liver (LiSyM, grant no. 031L0054). BC and H-MT are supported by the Deutsche Forschungsgemeinschaft (DFG, German Research Foundation) in a joint research project (CH 109/25 and TA 1583/1). UD is supported by the Deutsche Forschungsgemeinschaft (DFG, German Research Foundation) by grant no. DA 251/13-1 (SteaPKMod). MM is supported by the Carl-Zeiss-Stiftung by grant no. IKZ0563-2.8/738/2 and 5575/10-9.

## Conflict of Interest

The authors declare that the research was conducted in the absence of any commercial or financial relationships that could be construed as a potential conflict of interest.

## Publisher's Note

All claims expressed in this article are solely those of the authors and do not necessarily represent those of their affiliated organizations, or those of the publisher, the editors and the reviewers. Any product that may be evaluated in this article, or claim that may be made by its manufacturer, is not guaranteed or endorsed by the publisher.

## Abbreviations

ALPPS: associating liver partition and portal vein ligation for staged hepatectomy
AMPK: AMP-activated protein kinase
ARFI: acoustic radiation force impulse
ASL: arterial spin-labeling
CDK: cyclin-dependent kinase
CFD: computational fluid dynamics
ChE: cholinesterase
CT: computed tomography
CYP: cytochrome P450
DCE-MRI: dynamic contrast-enhanced MRI
DSC-MRI: dynamic susceptibility contrast MRI
DWI: diffusion weighted imaging
ECM: extracellular matrix
EGF: epidermal growth factor
FAK: focal adhesion kinase
FLR: future liver remnant
FOO: focal outflow obstruction
Gd-EOB-DTPA: gadolinium ethoxybenzyl-diethylenetriaminepentaacetic acid
GO: gene ontology
GS: glutamine synthetase
GSK3B: glycogen synthase kinase 3 beta
HBF: hepatic blood flow
HBS: hepatobiliary mebrofenin scintigraphy
HCC: hepatocellular carcinoma
HEF: hepatic extraction fraction
HGF: hepatocyte growth factor
HSC: hepatic stellate cells
HVPG: hepatic vein pressure gradient
ICG: indocyanine green
ICG-PDR: indocyanine green plasma disappearance rate
IVIM: intravoxel incoherent motion
JAM: junctional adhesion molecules
KEGG: kyoto encyclopedia of genes and genomes
LiMAx: Liver MAximum capacity test
LSEC: liver sinusoidal endothelial cells
MAPK: mitogen-activated protein kinase
MDRP2: multidrug resistance protein 2
MOR: model order reduction
MRE: magnetic resonance elastography
MRI: magnetic resonance imaging
MR: magnetic resonance
NAFLD: non-alcoholic fatty liver disease
NO: nitric oxide
OPS: orthogonal polarization spectroscopy
PBPK: physiologically based pharmacokinetic
PDFF: proton-density fat fraction
PET: positron emission tomography
PHx: partial hepatectomy
PI3K: phosphoinositide 3-kinase
PKC: protein kinase C
PK: pharmacokinetic
PVL: portal vein ligation
ROI: regions of interest
SBML: systems biology markup language
SPECT: single-photon emission-computed tomography
SWE: shear wave elastography
TAZ: WW domain-containing transcription regulator protein 1
TGF-β: transforming growth factor β
US: ultrasound
YAP: Yes-associated protein
ZO: zonula occludens proteins.
